# The effects of multimodal cocktail analgesic local injection in postoperative pain control after laminoplasty: A study protocol of a prospective randomized controlled trial

**DOI:** 10.1371/journal.pone.0324791

**Published:** 2025-06-13

**Authors:** Jaewan Soh, Hong-Sik Park, Won-Young Lee, Se-Hwan Park, Kyung-Chung Kang

**Affiliations:** 1 Department of Orthopaedic Surgery, College of Medicine, Hanyang University Guri Hospital, Hanyang University, Guri, Republic of Korea; 2 Department of Orthopaedic Surgery, College of Medicine, Kyung Hee University Hospital, Kyung Hee University, Seoul, Republic of Korea; National Trauma Research Institute, AUSTRALIA

## Abstract

**Background:**

Laminoplasty is the most widely used surgical technique for cervical spondylotic myelopathy. This surgery can cause severe postoperative pain; if not controlled, recovery or rehabilitation may be delayed. Therefore, effective control of postoperative pain is crucial. This randomized prospective study aims to evaluate the effects of a multimodal cocktail injection on postoperative pain and the efficacy of the protocol in patients undergoing posterior laminoplasty for cervical myelopathy.

**Methods:**

This single-center prospective randomized controlled trial focuses on patients diagnosed with cervical myelopathy or radiculopathy. This study will include patients aged 20–80 years who underwent laminoplasty. Participants will be divided into two groups: one group will receive a multimodal cocktail local injection during surgery and the other group will receive a local injection of normal saline only. The study is scheduled for a 3 month follow-up. The primary outcome measure will be the visual analog scale (VAS) score. Secondary outcome measures will be opioid and rescue analgesic consumption, time of initial analgesic requirement, adverse effects, and Japanese Orthopaedic Association (JOA) and neck disability index (NDI) scores.

**Results and conclusions:**

This is the first prospective randomized controlled trial to analyze the effects and safety of multimodal cocktail injections after cervical laminoplasty. Through this study, we anticipate that the demonstration of potential usefulness of multimodal cocktail analgesic injections in various aspects of spinal surgery, thereby this will provide a protocol for intraoperative cocktail injection.

**Trial registration:**

This trial was registered at the (https://www.clinicaltrial.gov), (NCT06113497) on 11/12/2023.

## Introduction

Laminoplasty is the most widely used surgical technique for cervical spondylotic myelopathy and offers significant advantages in preserving neck motion and relatively safe execution after surgery [1–3]. However, it involves dissection of the posterior neck muscles and bone procedures, contributing to substantial postoperative pain experienced by patients [3,4]. For patients undergoing surgery, the most significant concern is the pain experienced after the procedure, which can occasionally lead to cancellation or postponement of surgery [5,6]. When postoperative pain is not effectively managed, it negatively impacts recovery and rehabilitation [7–9], extends hospital stays, increases readmission rates, and escalates treatment costs [8,10]. Consequently, these effects can diminish patient satisfaction with treatment and hinder the patient’s willingness for subsequent interventions [11–13].

Therefore, effectively controlling postoperative pain is crucial, and maintaining optimal patient condition is a significant issue. Various options exist for postoperative pain control [14], but individual medications may have limitations due to their side effects [15–19]. The postoperative pain mechanism is multifactorial, prompting Kehlet and Dahl to introduce multimodal analgesia [11]. Although there may be several discrepancies between the fast progress in pain pathophysiology and the rather slow advances in providing optimal postoperative pain treatment, one important factor may be that more than 95% of the literature on postoperative pain treatment has considered unimodal treatment. Because total or optimal pain relief allowing normal function can not be achieved by a single drug or method, we should think of combined analgesic regimens or a multimodal approach to the treatment of postoperative pain. This involves maximizing the effectiveness of individual options and using various types of analgesics at appropriate dosages and routes of administration to achieve the best pain relief while minimizing side effects. By combining medications that act synergistically via different mechanisms at minimal doses, multimodal analgesia can minimize postoperative pain and enhance surgical outcomes. Applying this concept to surgery involves attempting pain control through localized administration of medications to the surgical site, which is widely utilized in various fields of orthopaedic surgery, especially joint replacement surgery [20–23]. In spinal surgery, studies have been conducted on the effects of local cocktail injections during lumbar surgery [24,25]. However, research on the effects of local injections of multimodal cocktail analgesics after cervical spine surgery is limited.

This randomized prospective study aims to evaluate the effects of multimodal cocktail therapy on postoperative pain and its utility in patients undergoing laminoplasty for cervical myelopathy. Through this study, we expected that the demonstration of potential usefulness of multimodal cocktail analgesic injections in various aspects of spinal surgery, thereby this will provide a protocol for intraoperative cocktail injection.

## Materials and methods

**Fig 1:**
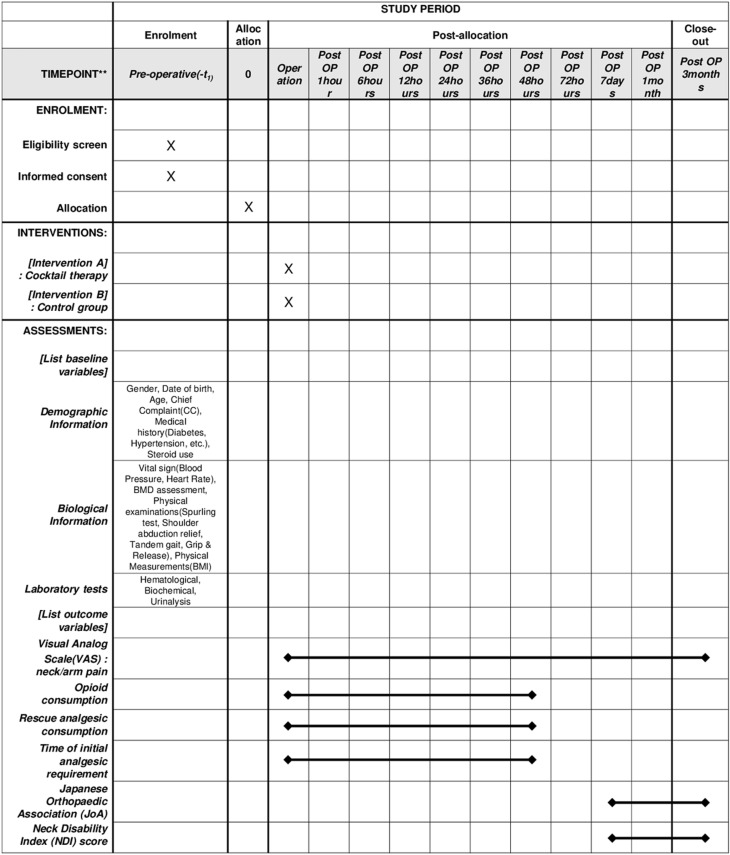
SPIRIT schedule of the schedule of enrolment, interventions, and assessments. This figure presents the SPIRIT (Standard Protocol Items: Recommendations for Interventional Trials) schedule outlining the timeline of participant enrollment, interventions, and assessments in the study. It provides an overview of the study design and key evaluation points.

This study is a registry-based randomized controlled trial (NCT06113497) at the (https://www.clinicaltrial.gov) and the protocol has been approved by the Institutional Review Board (IRB) of the Kyung-Hee University Hospital. All trial procedures are summarized in Fig 1.

### Study design

This single-center prospective randomized controlled trial focuses on patients diagnosed with cervical myelopathy or radiculopathy. All patients underwent laminoplasty performed by a single skilled surgeon. Participants will be divided into two groups: one in which an intraoperative cocktail injection (cocktail mixture) was administered and the other in which it was not, with patients consenting to participate prior to the procedure. To ensure technical soundness and minimize undisclosed flexibility, all procedures will follow a strictly standardized protocol, including block randomization (block size = 4) and a double-blind study design where both investigators and participants remain blinded to the treatment allocation.

### Participants

All patients visiting Kyung Hee Medical Center underwent 4-level laminoplasty at C3, C4, C5, and C6. After the initial screening, suitable participants were selected, and surgery was performed under the supervision of Professor K.C.K.

### Inclusion criteria

Inclusion criteria consists of patients i) diagnosed with cervical myelopathy or radiculopathy and scheduled to undergo laminoplasty; ii) reported a preoperative upper limb pain (visual analog scale [VAS]), neck pain (VAS), Japanese Orthopaedic Association (JOA) score, and Neck Disability Index (NDI) score; iii) adults aged 20 years and above but below 80 years capable of articulating their own pain or functional abnormalities; and iv) having a cognitive function at a level that enables them to comprehend and adhere to the study procedures.

### Exclusion criteria

Exclusion criteria consists of patients i) with previous surgical treatment of the cervical spine; ii) aged 20 years or younger, pregnant individuals, or those with the potential for pregnancy; iii) with hypersensitivity reactions to mixed medications used in cocktail therapy (morphine 5 mg, ropivacaine hydrochloride 150 mg, triamcinolone acetonide 40 mg, epinephrine 1 mg, ketorolac tromethamine 60 mg, cefotiam hydrochloride 1 g); and iv) who lack the capacity for medical consent or are unable to communicate effectively in a medical context.

### Randomization

**Fig 2:**
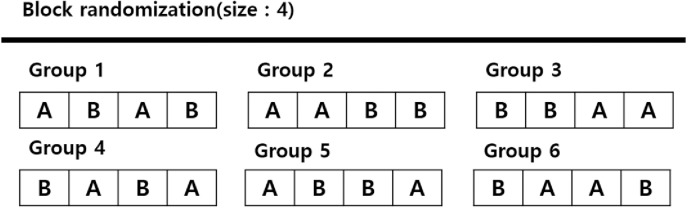
Block randomization. This figure illustrates the block randomization method used in the study. Participants were randomly assigned in blocks of four to ensure balanced allocation between groups and enhance the reliability of the study.

The participants were randomized using the block randomization method (block size = 4; Fig 2). Randomly assigned participants will be evaluated for efficacy and safety through outpatient visits at 4 and 12 weeks after surgery, in addition to the hospitalization period.

### Blinding

**Fig 3:**
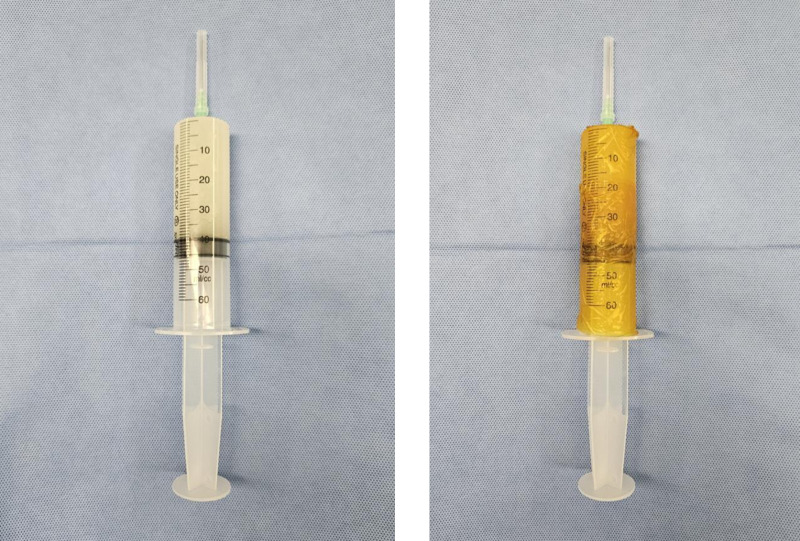
The blinding method using Iovan. The cocktail drug exhibits an opaque white color, and blinding was performed using Iovan. This figure demonstrates the blinding method using Iovan. The multimodal cocktail drug appears as an opaque white solution, and Iovan was used to cover the syringe contents, ensuring that both researchers and participants remained blinded to the assigned group.

Cocktail analyses and an equivalent volume of sterile saline will be prepared independently in a sterile manner in an operating room. Then, mark them as A and B, respectively, and inject one of the two into the investigator to match the blocked randomization. The investigator and the patient will not know what A and B are and will be kept in a double-blind state regarding group assignment. The syringe will be covered with Iovan, and the surgeon will be blinded to the contents (Fig 3). Data will be collected by research personnel who will be blinded to the group allocation. After study completion, the data will be analyzed by a statistical specialist unaware of the group assignments. Clinical trial drugs will be managed, distributed, and returned using random allocation codes (or clinical trial drug codes), which will be maintained by a code manager (blind coordinator).

### Interventions

All patients will be randomized into either the cocktail or control groups, and will be scheduled to undergo cervical laminoplasty. The patients in the cocktail group will receive a multimodal cocktail analgesic injection into the perimuscular area immediately before wound closure. The patients in the control group will receive the same volume of saline via injection using the same method. A single surgeon will perform all the procedures.

### Cocktail analgesic regimen

Morphine 5 mg, ropivacaine 150 mg, tramadol 40 mg, epinephrine 1 mg, ketorolac 60 mg, and ketamine 1 g will be mixed with normal saline to prepare a total volume of 60mL. This regimen was determined as a cocktail regimen because Teng et al. [26] mixed ropivacaine, epinephrine, ketorolac, and normal saline for local infiltration in total knee arthroplasty, Kim et al. [27] added morphine, Mohamed et al [28]. added ketamine, and Mitra et al. [29] added tramadol and achieved good results.

### Cocktail analgesic injection group (cocktail group)

During wound closure, 60mL of a cocktail of analgesics will be injected into the surgical site’s deep fascia and the muscular layer. Postoperative pain control will involve administering fentanyl intravenously (IV) as a bolus of 50 mcg immediately after surgery, followed by patient-controlled analgesia (IV-PCA) with a basal infusion of 0.3 mcg/kg/h, which can be sustained for 48 h. An infuser with a 15-min lockout time setting will be used, allowing for bolus administration of 0.075 mcg/kg.

### Sterile saline injection group (control group)

During wound closure, 60 mL of normal saline will be injected into the surgical site’s deep fascia and muscular layers. Postoperative pain control will follow the same approach as in the cocktail group using the methods described earlier.

### Perioperative management

For preoperative pain management, celecoxib 200 mg and pregabalin 75 mg were administered orally once after dinner the day before surgery, and no intraoperative pain management was performed.

On the morning of the surgery, mix 60 mg of nefopam and 1000 mg of acetaminophen in 1 L of fluid and administrate to drop it intravenously, and apply one patch each of buprenorphine 5 mg and fentanyl 2.1 mg before going to the operating room.

Surgery will be performed under standard general anesthesia. Intraoperative fluid management will also be standardized according to the surgical plan, using a balanced crystalloid and hydroxyethyl starch colloid. After surgery, the IV-PCA infuser will be connected and removed after 48 h.

IV-PCA is prepared by mixing fentanyl 0.8 ~ 1 mg and ramosetron 0.6 mg.

Following surgery, patients will be monitored in the post-anesthesia care unit according to standardized criteria. They will be assessed for their condition and transferred to a hospital room. Until 24 h postoperatively, careful evaluation of the patient’s condition and promotion of lung function recovery through deep breathing and coughing techniques will be conducted.

The time of the first bedside mobilization after surgery and additional analgesic consumption will be recorded. The patient’s condition will be continuously monitored until discharge. The patient will be discharged on the 7th day after surgery if no complications arise. Regular outpatient follow-up visits will be scheduled for symptom assessment 4 and 12 weeks after surgery.

### Postoperative basal analgesia

The experimental and control groups will undergo various pain management strategies via different postoperative routes to ensure pain control (Table 1).

**Table 1:** Postoperative basal analgesia.

	Postop 1 day	Postop 2 days – discharge
**Fluid**	Nefopam 60 mg + 0.9% Normal saline	Nefopam 60 mg + 0.9% Normal saline
	Oxycodone 10 mg + 0.9% Normal saline	
**PO medication**	Traspen tab (Tramadol 37.5 mg, Acetaminophen 325 mg) 1T bid	Traspen tab (Tramadol 37.5 mg, Acetaminophen 325 mg) 1T bid

### Rescue analgesia

After surgery, patients who report pain of 4 or more on the VAS will receive a 50 mg intramuscular injection of tramadol (tramadol hydrochloride). Patients who report pain of 6 or more on the VAS will receive a 25 mg intramuscular injection of pethidine (pethidine hydrochloride). This will serve as a postoperative pain assessment scale, and medication administration time and frequency will be recorded until discharge.

### Outcome assessments

#### Data capture.

Before enrolling study participants,

1) Collect demographic information of study participants (gender, date of birth, age), chief complaint (CC), medical history (diabetes, etc.) and steroid use.
2) Perform vital sign measurements (blood pressure, heart rate), BMD assessment, physical examinations (Spurling test, shoulder abduction relief, tandem gait, grip & release), and physical measurements (BMI).
3) Conduct laboratory tests (hematological, biochemical, urinalysis).

The effects of injecting cocktail analgesics through the subcutaneous tissue during the laminoplasty procedure during wound closure on postoperative pain will be investigated. Postoperative pain will be assessed at 1, 6, 12, 24, 36, 48, and 72 h; 7 d; and 1 and 3 months. When we follow up postoperative patients in outpatients, we usually do so at 2 weeks, 4 weeks, and 3 months after surgery. Accordingly, we performed evaluations at 1 and 3 months after surgery. VAS scores, opioid consumption, and frequency of other analgesic use will be recorded to assess and compare pain levels between the two groups.

**Fig 4:**
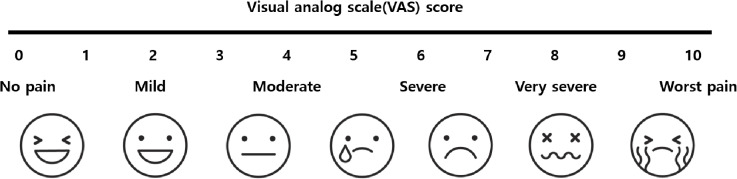
Visual analog scale(VAS). This figure displays the Visual Analog Scale (VAS) used for postoperative pain assessment. The VAS is a quantitative tool for evaluating pain levels, and in this study, it was used to track pain progression from 1 hour postoperatively to 3 months after surgery.

1) VAS score (Fig 4)
2) Opioid consumption
3) Rescue analgesic consumption
4) Time of initial analgesic requirement
5) Adverse effects
6) JOA and NDI scores (Tables 2 and 3).

**Table 2:** Japanese Orthopaedic Association (JOA) score.

A. Motor function	
Upper extremity	
0	Unable to feed onself
1	Can manage to feed oneself with spoon or fork but not with chopsticks
2	Either chopsticks feeding or writing is possible but not practical
3	Either chopsticks feeding or writing is clumsy but practical
4	Normal
Lower extremity	
0	Unable to stand & walk by any means
1	Unable to walk without cane or other support on a level
2	Walks independently on a level but needs support on stairs
3	Capable of fast but clumsy walking
4	Normal
**B. Sensory function**	
Upper extremity	
0	Apparent sensory disturbance
1	Minimal sensory disturbance
2	Normal
Lower extremity	
0	Apparent sensory disturbance
1	Minimal sensory disturbance
2	Normal
Trunk	
0	Apparent sensory disturbance
1	Minimal sensory disturbance
2	Noramal
**C. Bladder function**	
0	Urinary retention or incontinence
1	Sense of retention or dribbling or thin stream or incomplete continence
2	Urinary retardation or pollakiuria
3	Normal
	**Total score**

**Table 3:** Neck Disability Index (NDI) score.

1. Pain Intensity		6. Concentration	
No pain at the moment	0	I can concentrate fully when I want with no difficulty	0
Very mild at the moment	1	I can concentrate fully when I want to with slight difficulty	1
Moderate at the moment	2	I have a fair degree of difficulty concentrating when I want to	2
Fairly severe at the moment	3	I have a lot of difficulty concentrating when I want to	3
Very severe at the moment	4	I have a great deal of difficulty concentrating when I want to I cannot concentrate at all	4
The worst pain imaginable at the moment	5	I cannot concentrate at all	5
**2. Personal Care**		**7. Work**	
I can look after myself normally without causing extra pain	0	I can do as much work as I want	0
I can look after myself, but it causes extra pain	1	I can only do my usual work, but no more	1
It is painful to look after myself and I am slow and careful	2	I can do most of my usual work, but no more	2
I need some help but manage most of my personal care	3	I cannot do my usual work	3
I need help every day in most aspects of sef-care	4	I can hardly do any work at all	4
I do not get dressed; I wash with difficulty and stay in bed	5	I cannot do any work at all	5
**3. Lifting**		**8. Driving**	
I can lift heavy weights without extra pain	0	I can drive my car without any neck pain	0
I can lift heavy weights, but it gives me extra pain	1	I can drive my car as long as I want with slight neck pain	1
Pain prevents me from lifting heavy weights off the floor, but I can manage if they are conveniently positioned	2	I can drive my car as long as I want with moderate neck pain	2
Pain prevents me from lifting heavy weights, but I can manage light-to-medium weights if they are conveniently positioned	3	I can’t drive my car as long as I want because of moderate neck pain	3
I can lift very light weights	4	I can hardly drive at all because of severe neck pain	4
I cannot lift or carry anything at all	5	I can’t drive my car at all	5
**4. Reading**		**9. Sleeping**	
I can read as much as I want with no neck pain	0	I have no trouble sleeping	0
I can read as much as I want with slight neck pain	1	My sleep is slightly disturbed (less than 1 hour sleepless)	1
I can read as much as I want with moderate neck pain	2	My sleep is mildly disturbed (1–2 hours sleepless)	2
I can’t read as much as I want because of moderate neck pain	3	My sleep is moderately disturbed (2–3 hours sleepless)	3
I can’t read as much as I want because of severe neck pain	4	My sleep is greatly disturbed (3–5 hours sleepless)	4
I cannot read at all	5	My sleep is completely disturbed (5–7 hours sleepless)	5
**5. Headaches**		**10. Recreation**	
I have no headaches at all	0	I am able to engage in all my recreation activities with no neck pain	0
I have slight headaches that come infrequently	1	I am able to engage in all my recreation activities with some neck pain	1
I have moderate headaches that come infrequently	2	I am able to engage in most, but not all, of my usual recreation activities because of neck pain	2
I have moderate headaches that come frequently	3	I am able to engage in a few of my usual recreation activities because of neck pain	3
I have severe headaches that come frequently	4	I can hardly do any recreation activities because of neck pain	4
I have headaches almost all of the time	5	I can’t do any recreation activities at all because of neck pain	5
**Total score**			

2)-4) items are planned to be measured during the one-week hospitalization period, and VAS, JOA, NDI, adverse effects will be measured at the 7-day hospitalization period and at 1 and 3 months of outpatient follow-up.

### Primary outcome measures

The primary outcome will involve recording the average pain scores around the surgical site, using the VAS for neck, arm pain. These scores will be documented during the hospitalization period up to 7 d after surgery and at 1 and 3 months postoperatively, following the scheduled assessment program.

### Secondary outcome measures

The total use of IV-PCA up to 48 h postsurgery will be recorded. The number of IV-PCA push attempts up to 48 h postsurgery will be documented. Additional analgesic consumption (Tramadol IM, Pethidine IM) beyond PCA will be measured. The time of the initial requirement and the corresponding VAS score will be recorded. Adverse effects, including nausea and vomiting, are documented. The JOA and NDI scores will be recorded 7 d and 1 and 3 months after surgery.

### Sample size calculation

The sample size was determined using a power analysis (α = 0.05, 2-sided; power = 80%). This analysis was based on a pilot study conducted by Kraiwattanapong et al. [25], in which the mean VAS score in the experimental group was set at 3, and the mean VAS scores in the control group were set at 3 and 1, respectively. Assuming a 20% dropout rate for 50 participants (25 in each group), a total sample size of 60 was deemed necessary.

### Discontinuation criteria

Maintaining enrolled patients until the end of the study is a critical concern, and minimizing dropout rates is essential. However, in the event of serious adverse effects associated with each agent in the cocktail injection, the study of the affected patients will be discontinued to ensure patient safety. Similarly, if significant adverse effects related to patient-required analgesics occurred after surgery, the patients will be excluded from the study to prioritize safety. Furthermore, even in cases where such issues do not arise, patients requesting discontinue participation in the study will be excluded.

If patients drop out or are lost during the study, the robustness of the analysis will be improved by multiple imputations or the last observation carried forward method, as missing data may affect the results over time.

### Adverse event reporting and safety

Specific documentation will be maintained for any significant adverse effects during the study, and regular evaluations will be conducted in this regard. The assessment panel will consist of at least two evaluators, and based on the evaluation results, reports will be submitted to the Institutional Review Board (IRB) committee overseeing the study.

### Data analysis

For continuous variables, an analysis of variance with an unpaired t-test will be used for variables with normality and the Wilcoxon rank-sum test for variables without normality. Categorical variables will be assessed using the chi-square test and compensated using Fisher’s exact test. Repeated measures analysis to evaluate trends in pain scores over time will be performed. All statistical calculations will be performed using SPSS software (version 25.0; IBM Corp., Armonk, NY, USA). Statistical significance is set at P < 0.05.

### Ethics approval, monitoring, audit and consent to participate

All procedures will be performed in compliance with our department’s standards and the Declaration of Helsinki, and written informed consent will be obtained from all participants. This study was approved by the Institutional Review Board (IRB) of the Kyung-Hee University Hospital. The collected data will be audited and monitored by the IRB. Researchers will conduct a mid-term report of the study at the time specified by the Institutional Review Board (IRB).

### Data recording and confidentiality

Documents related to clinical research are transferred to the institution’s document custodian upon the completion of the study and are preserved for a period of 3 years.

### Ancillary and post-trial care

If any harm occurs while participating in this clinical trial, you will receive treatment in accordance with the hospital’s standard medical procedures.

## Discussion

This study aims to evaluate the analgesic effects of local injection of a multimodal cocktail analgesic following cervical laminoplasty. Spinal surgery presents challenges in postoperative pain management, making effective pain control paramount for improving patient satisfaction and facilitating smooth postoperative rehabilitation, ultimately enabling a quicker return to normal daily activities [30–32]. Until now, numerous efforts have been made to manage postoperative pain adequately; however, a consensus protocol has yet to be established. Various methods are used in practice. Since the initial discovery of the efficacy of local anesthetic infiltration in lumbar spine surgery by Mullen et al. [33], this approach to pain management has been widely adopted. In 2017, Perera et al. [24] conducted a meta-analysis of 11 prospective randomized controlled trials on intramuscular local analgesic injections after lumbar spine surgery. Their findings suggested its effectiveness in reducing postoperative opioid demand, prolonging the time to the first analgesic requirement, and decreasing VAS scores in the first hour after surgery. However, research on the use and efficacy of this approach in cervical spine surgery is limited.

### Pain alleviation

Among the various methods used to control pain after spinal surgery, opioids are the most widely used. Surgical trauma triggers an inflammatory response that leads to the expression of opioid receptors in inflamed tissues [34]. Opioid receptors are expressed within hours of surgical trauma and provide regional analgesia. In addition, they play a role in transmitting afferent sensory inputs to the central nervous system, thereby inducing rapid antinociceptive effects [35,36]. Therefore, administering morphine through local infiltration [37] is anticipated to be highly effective in alleviating postoperative pain. In addition to local infiltration, morphine is also employed for postoperative pain control through various routes, such as epidural catheter-based morphine delivery, IV-PCA with morphine, and intrathecal morphine administration [38]. Despite these options being widely used, a consensus on the most effective method has not yet been reached due to ongoing controversies and complications associated with each approach [38–40].

Tramadol possesses both analgesic as well as peripheral local anesthetic activity [29]. Studies on postoperative pain utilizing patient-controlled analgesia have concluded that tramadol is as equipotent as pethidine, Hancı et al. [41] supported that tramadol can be used for wound infiltration with no side effect on wound healing.

### Reducing inflammatory response

Postoperative inflammatory responses increase surgical site pain [37–39] and are associated with various complications, such as cognitive impairment and delirium. These complications can negatively affect surgical outcomes [42–44]. Therefore, reducing postoperative inflammatory responses is essential for pain control and can contribute to achieving satisfactory surgical results.

The cocktail of analgesics used in the present study will include ketorolac. Ketorolac is a nonsteroidal anti-inflammatory drug (NSAID) that inhibits the production of COX-1 and COX-2, enzymes that generate proinflammatory mediators such as prostaglandins. This reduced the inflammatory response. Peripheral prostaglandins sensitize pain receptors (nociceptors) and increase pain signaling in the brain [45]. Including NSAIDs, like ketorolac, can reduce inflammatory responses and desensitize nociceptors, alleviating pain.

The steroid component of the multimodal cocktail of analgesics inhibits the synthesis of phospholipase A2, suppresses immune cell activity, and reduces the production of inflammatory mediators and cytokines [46]. Steroids are crucial in decreasing inflammatory responses, particularly by inhibiting prostaglandin production. According to Ikeuchi et al. [47], the anti-inflammatory effect of steroids in local analgesic infiltration reduces local inflammatory responses due to surgical trauma and has systemic anti-inflammatory effects.

Moreover, studies like that by Xiong et al. [48] involving patients who underwent hip arthroplasty following periarticular multimodal anesthetic injection demonstrated significantly lower levels of inflammatory markers (C-reactive protein, erythrocyte sedimentation rate, interleukin[IL]-1β, IL-6) and VAS scores during the postoperative period. This finding suggests potential benefits regarding surgical outcomes from local analgesic infiltration, a result applicable to cervical laminoplasty, a motion-preserving surgery. This evidence supports the use of local injections to improve surgical outcomes of cervical laminoplasty.

### Local anestghesia and ketamine

Most previous studies used bupivacaine as one of the components of the cocktail. Even though ropivacaine shares some pharmacokinetic characteristics with bupivacaine, it has a longer half-life, lower toxicity to the heart and central nervous system, and a higher tolerability in patients [49].

The peripheral analgesic effect of ketamine may be easily explained by blocking sodium and potassium currents in peripheral nerves [50], the combination of ketamine with other peripheral-acting analgesics may produce synergistic pain relief and obviate the side effects that often result from systemic therapy with single drugs in higher doses [51].

### Prolongation of the effect of the local agents

Including epinephrine in the multimodal analgesic cocktail used in this study serves a specific purpose. Epinephrine’s α-adrenergic effects are used to achieve certain benefits. Epinephrine can reduce the absorption of local agents by inducing vasoconstriction of the vessels surrounding the surgical site. This prolongs each agent’s local effects and improves pain control. Furthermore, the vasoconstrictive action of epinephrine helps minimize postoperative bleeding and hematoma formation, potentially leading to improved surgical outcomes. By utilizing the vasoconstrictive properties of epinephrine, this study aims to enhance the efficacy of local injections of multimodal cocktail analgesics and promote better pain management while reducing potential postoperative complications related to bleeding and hematoma [52].

### Prevention of surgical site infection

Steroids can influence a patient’s immune response and increase the risk of infection [53]. While many reports in the context of arthroplasty have suggested that using steroids for local anesthetic infiltration does not significantly increase the risk of postoperative infection [52,54–56], some opposing findings also exist [57,58]. This underlines the ongoing caution and considerations surrounding the possibility of surgical site infections. Using a multimodal cocktail analgesic regimen prepared by nurses in the operating room introduces the possibility of contamination and the associated infection risks. Prophylactic antibiotics, specifically cephalosporins, have been included in the regimen to mitigate this risk.

Cervical laminoplasty is indeed a surgical procedure that often involves significant postoperative pain. A study by Hida et al. [38] indicated that patients who received diclofenac suppositories after cervical laminoplasty reported VAS scores of 5.1 at postoperative 4 h postoperatively and 4.8 at 24 h postoperatively. Furthermore, self-assessment of postoperative pain revealed that 77% of the patients reported “severe pain” 1 week postoperatively. Similarly, Yukawa et al. [59] found that patients undergoing cervical laminoplasty reported VAS scores of 50 on postoperative day 1, 39.5 on postoperative day 3, and 30.4 at postoperative 1 week. According to the literature, cervical laminoplasty is generally associated with significant postoperative pain, with VAS scores typically exceeding five points. Therefore, appropriate postoperative pain control is essential. Results of this study are expected to expand the scope of pain management in cervical spine surgery using local analgesic infiltration, thereby providing relief to patients experiencing significant postoperative pain.

## Conclusion

This is the first prospective randomized controlled trial to analyze the effects and safety of multimodal cocktail injections after cervical laminoplasty. Through this study, we anticipate the demonstration of potential usefulness of multimodal cocktail analgesic injections in various aspects of spinal surgery, thereby offering valuable insights and possibilities.

## Supporting information

S1 File SPRIRT checklist. (DOC)

S2 File Study protocol(Korean version). (DOCX)

S3 File Study protocol(translated to Eng). (DOCX)

## Abbreviations

VAS: Visual analog scale
JOA: Japanese orthopaedic association
NDI: Neck disability index
IV: Intravenously
PCA: Patient-controlled analgesia
IRB: Institutional Review Board
NSAID: Nonsteroidal anti-inflammatory drug.
