# Supplementary material for: The effects of multimodal cocktail analgesic local injection in postoperative pain control after laminoplasty: A study protocol of a prospective randomized controlled trial
Source: PLoS One. 2025 Jun 13;20(6):e0324791. doi: 10.1371/journal.pone.0324791 (PMC12165372; doi:10.1371/journal.pone.0324791)
Supplement: S2 File — (DOCX) [file pone.0324791.s002.docx]

【연구 계획서】

1. 연구명칭

­

A prospective study on the comparison of postoperative pain according to the use of cocktail therapy in laminoplasty

(후궁성형술에서의 cocktail therapy사용에 따른 수술 후 통증 비교에 대한 전향적 연구)

2. 연구기관명 및 주소

Department of Orthopaedic Surgery, Kyung Hee University Medical Center, Kyunghee University School of Medicine, 23, Kyungheedae-ro Dongdaemun-gu Seoul, Republic of Korea 130-872

TEL: 82-2-958-8346

E-mail: futurespine@gmail.com

3. 연구책임자, 담당자 및 공동연구자의 성명 및 직명

연구책임자 및 담당자 강경중 Kang Kyung-Chung, MD (교수)

연구담당자 이원영 Lee Won-Young, MD(전임의)

연구담당자 박민정 Park Min-Jeong (CRC)

4. 연구 목적 및 배경

Laminoplasty는 경추 척수증에서 가장 널리 사용되고 있는 수술방법으로 수술 후 neck motion을 보존할 수 있고 비교적 안전하게 진행할 수 있다는 측면에서 큰 장점이 있다.(1-3) 하지만 수술 중 posterior neck muscle을 dissection 해야 하며 bone procedure를 동반하는 과정을 포함하고 있어 수술 후 환자가 느끼는 통증은 큰 편에 속한다.(3, 4) 수술을 받는 환자들에게 있어서도 가장 큰 걱정은 수술 그 자체보다 수술 후 느끼게 될 통증으로 이로 인해 종종 수술 직전의 취소 혹은 수술 연기로 이어지는 경우도 있다.(5, 6) 수술 후 느끼는 통증이 잘 조절되지 않을 경우 수술 후 재활의 진행에도 부정적인 영향을 끼치게 된다.(7-9) 결과적으로 이러한 효과는 입원기간의 연장, 재입원율의 증가 및 치료 비용 증가에 기여하게 된다.(8, 10) 무엇보다 수술 후 통증 조절을 적절하게 하지 못할 경우 환자의 만족도를 떨어뜨리게 되고 향후 추가 치료가 필요한 경우 이에 대한 환자의 의지를 충분히 채우지 못하게 되는 주된 원인이 되기도 한다.(11-13) 따라서 수술 후 통증에 대한 충분한 조절이 필요하며 환자가 적절한 컨디션을 유지할 수 있도록 하는 것은 중요한 이슈가 된다. 여러가지 수술 후 통증 조절을 위한 옵션들이 있지만 개별 약제들이 가진 side effect로 인해 적절히 사용할 수 없는 경우들이 있다.(14-18)

수술 후 통증의 기전은 multifactorial 한 것으로 알려져 있어 Kehlet and Dahl(11)등은 multimodal analgesia를 통한 통증 조절의 개념을 소개하였다. 이는 개별 옵션들의 효율성을 극대화하는 것으로 여러 종류의 진통제를 적절한 용량으로 사용하고 약의 투약 루트를 다양화하여 최대의 진통 효과를 얻고 부작용을 최소화할 수 있다는 것이다. 이것은 개별 약제들이 서로 다른 메커니즘에 따라 작용하여 최소한의 용량으로 synergy를 이룰 수 있도록 한다는 것으로, 이러한 multimodal analgesia는 수술 후 통증을 최소화하고 수술의 결과를 향상시키는데 도움을 줄 수 있을 것이다. 이러한 개념을 적용하여 수술 중 수술 부위에 국소적인 약제 투여를 통해 통증 조절을 시도하는 것은 정형외과 수술 각 분야, 특히 인공관절 수술 분야에서 널리 이용되고 있으며(19-22) 척추 수술에서는 요추 수술 중 시행한 국소 부위 cocktail injection의 효과에 대한 연구가 많이 수행되었다.(23, 24) 하지만 경추 수술 후 Multimodal cocktail analgesic local injection의 효과에 대한 연구는 일부에서 제한적으로 이루어진 실정이다.(25, 26) 이에 관해 본 연구에서는 경추척수증 혹은 경추신경근증이 있는 환자 중 후궁성형술을 받은 환자들을 대상으로, cocktail therapy 사용에 따른 수술 후 통증에 대한 평가와 그에 따른 유용성에 대해 평가해보고자 한다.

**목적**: 이에 관해 본 연구에서는 경추척수증 혹은 경추신경근증이 있는 환자 중 후궁성형술을 받은 환자들을 대상으로, cocktail therapy 사용에 따른 수술 후 통증에 대한 평가와 그에 따른 유용성에 대해 평가해보고자 한다.

5. 연구대상자 선정기준

**Inclusion criteria**

- 본원에 내원한 경추척수증 혹은 경추신경근증이 동반된 환자 중 후궁성형술을 시행 받을 예정인 자..

- 수술 전 상지 통증(VAS), 목 통증(VAS), JOA, NDI score 기록 결과를 가진 환자를 대상으로 함

- 20세 이상 80세 미만의 성인으로 본인의 통증이나 기능 이상을 표현할 수 있는 상태인 환자

- 본 연구 수행 절차를 이해하고 순응할 수 있는 수준의 인지 기능을 갖고 있는 환자

**Exclusion criteria**

- 본원에 내원한 경추척수증 혹은 경추신경근증이 동반된 환자 중 이전에 경추 부위에 수술적 치료를 받은 환자

- 20세 이하의 청소년이나 임산부

- Cocktail therapy 의 혼합 약제에 대한 과민성 반응이 있는 경우.

(Morphine 5mg, ropiva 150mg, tamceton 40mg, epinephrine 1mg, ketocin 60mg, jetiam 1g)

- 의학적으로 동의 능력이 없거나 의사소통이 불가능한 환자

6. 방법 및 평가

**<연구 방법>**

단일기관, 전향적, 무작위성 대조연구법으로 본원에서 경추척수증, 혹은 신경근증으로 진단받고 수술은 숙련된 single operator에 의해 수행된 후궁성형술의 수술적 치료를 시행하는 환자를 대상으로 사전동의 하에 intraoperative cocktail injection(cocktail 배합)를 시행한 군과 시행하지 않은 군으로 나누어 평가를 한다.

**<참여자>**

연구 대상자는 경추척수증 및 경추신경근증으로 인해 후궁성형술이 필요한 환자 중, 연구자 및 연구담당자의 충분한 사전설명 및 사전동의 하에 선정된다.

시험단계: 4상 / 시험기간: IRB 승인일로부터 2025년 9월 30일까지

**<무작위 배정>**

Random allocation은 block randomization 방법에 따라 시행예정(block size 4)


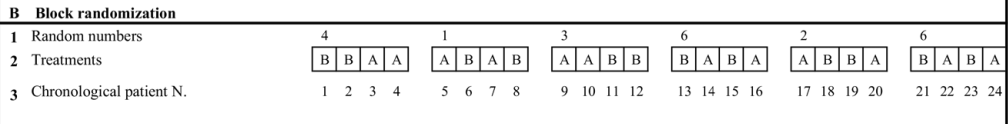


무작위 배정된 시험대상자는 입원 기간 및 수술 후 4주 후 외래 방문을 통해 유효성 및 안정성을 평가받는다.

**<Interventions>**

모든 환자는 randomized 되어 cocktail group과 control group 중 한 곳에 속할 것이다. cocktail group에 속한 환자들은 multimodal cocktail analgesic을 수술 후 wound closure 직전 perimuscular area에 injection을 받을 것이며 Control group의 환자들은 같은 용량의 saline을 동일한 방식으로 injection 받게 될 것이다. 모든 과정은 숙련된 single surgeon에 의해 진행될 것이며 모든 환자는 Cervical laminoplasty 수술을 받을 예정이다.

**<Blinding>**

Cocktail analgesics 또는 이에 상응하는 양의 saline은 무균 방식으로 독립적으로 수술실에서 준비한다. 집도의와 환자 모두 그룹 할당에 대해 double-blind 상태를 유지하고 데이터는 그룹 할당에 blind된 연구원이 수집할 예정이며 연구 종료 후 data는 그룹할당 정보 없는 통계전담 연구원이 분석할 예정이다.

**<이중눈가림에 대한 방법>**

본 임상시험에서는 과학적이고 정확한 결과를 산출하기 위해 투여기간 동안 외형상으로 구분이 불가능하도록 동일한 제형 및 성상의 위약대조 방법의 이중눈가림을 수행한다.

이 를 통해 시험대상자가 어느 군에 배정되었는지 시험자 및 시험대상자 모두가 구별할 수 없도록 한다. 눈가림은 본 시험의 완결성(integrity)에 매우 중요한 요소로, 배정군 정보는 임상시험 데이터 베이스가 잠금(locking), 보관될 때까지 시험자는 물론 코드 관리자를 제외한 모든 사람들에게 눈가림 상태로 유지될 것이다. 중대하고 예상하지 못한 이상반응(Suspected unexpected serious adverse reaction,SUSAR)을 평가, 보고하는 과정에서 눈가림이 해제되는 경우가 발생할 수 있으므로 , 임상시험에 참여하는 코드관리자(unblind담당자) 외 연구자들은 눈가림 유지를 위하여 SUSAR 평가, 보고 업무에 관여하여서는 안된다. 임상시험 의약품은 무작위배정 코드(또는 임상시험용 의약품 코드)로 관리, 배부, 반납될 것이며, 무작위배정 코드는 코드관리자(unblind담당자)가 관리한다. 육안으로 확인 시 시험약관 대조약의 성상이 동일하여 구분되지 않으며, 환자가 누워 있는 상태에서 임상시험용 의약품이 투여되며, 독립된 투여자에 의해 다루어지므로 환자의 눈가림이 가능하다 .

칵테일요법은 약제 혼합 시 불투명한 흰색의 색상을 나타내고, 대조약인 생리식염수는 투명하다. 여기에 아이오반을 붙여서 눈가림을 시행한다.


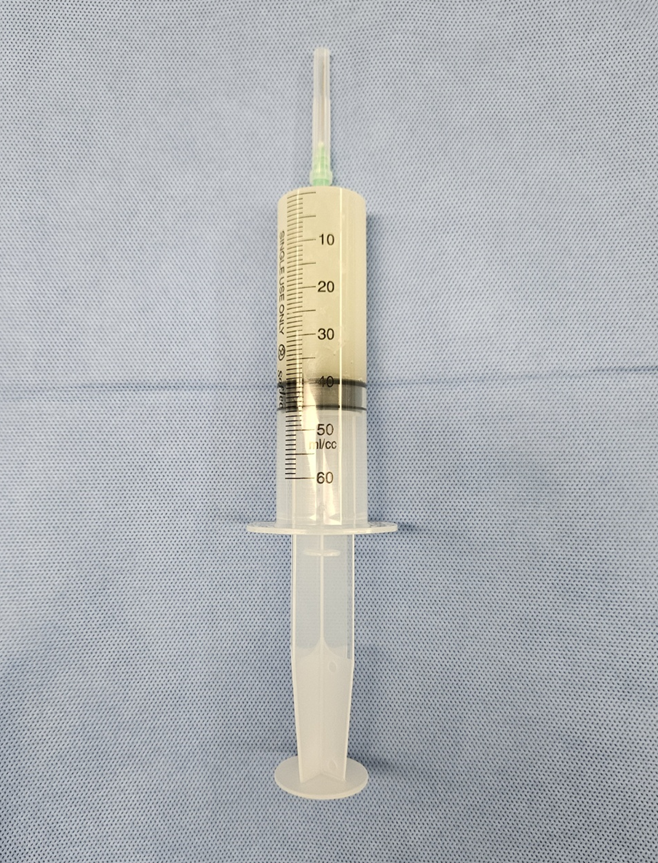

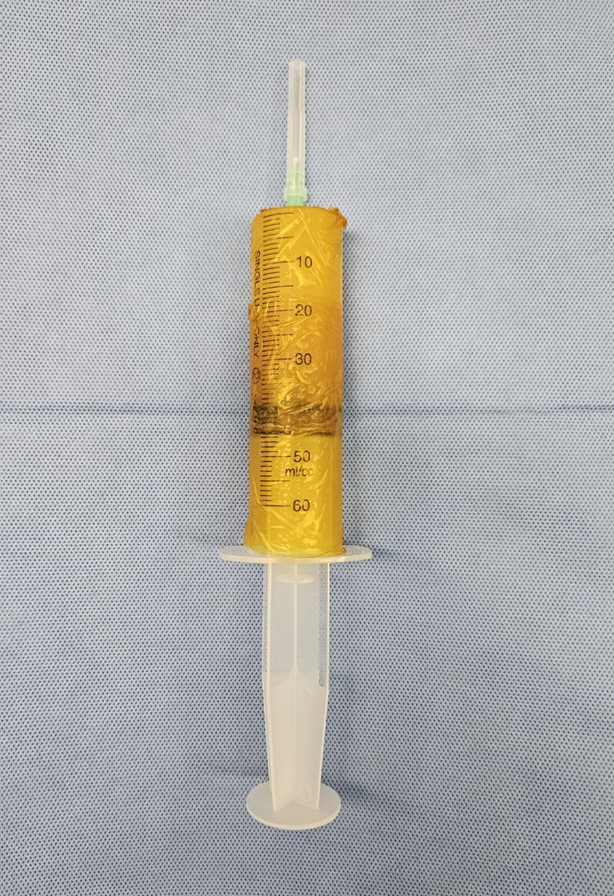


* 눈가림 인력 추가

blind 담당자 : PI 강경중 , CRC박민정

unblind 담당자 : sub-I 이원영 ( IP관리 및 투여 )

**<Cocktail analgesic injection group(cocktail group)>**

- **Cocktail therapy regimen**

Morphine 5mg, ropiva 150mg, tamceton 40mg, epinephrine 1mg, ketocin 60mg, jetiam 1g를 normal saline 섞어 total 40ml injection.

- 실험군 : wound closure 시 cocktail therapy를 deep fascia 및 muscular layer에 injection 한 group
- 대조군 : wound closure 시 normal saline 40ml injection..

실험군, 대조군 모두 수술 후 IV-PCA를 사용한다.

수술 후 통증 조절은 수술 직후 fentanyl iv bolus 50mcg inj 후 IV-PCA로 하며 basal infusion 0.3mcg/kg/h로 48시간 지속가능, 15min lock out time setting 하에 bolus 0.075mcg/kg 투약 가능한 infuser를 이용할 예정이다. 적용 후 48시간 이후 제거하며 IV-PCA를 처음 사용할 때까지 걸리는 시간, 사용량을 비교한다.

<**Sterile saline injection group(control group)>**

wound closure 시 normal saline 40ml 를 deep fascia와 muscular layer에 주사할 예정이다. 수술 후 통증 조절은 cocktail group과 동일한 방식으로 사용할 예정이다.

**<** **Perioperative management>**

수술은 표준화된 방법의 전신 마취 하에 진행될 예정이다. 수술 중 fluid management도 표준화된 수술 중 계획에 따라 수행될 예정으로 balanced crystalloid와 hydroxyethyl starch colloid를 적용한다. 수술 후에는 IV-PCA infuser를 연결할 것이고 수술 후 48시간에 제거할 예정이다. 수술 후에는 마취회복실에서 모니터한 후 표준화된 기준에 따라 환자 상태 평가 후 병실로 이동할 것이며 수술 후 24시간까지 신중하게 환자 상태 평가 및 폐 기능 회복을 위한 Deep breathing & coughing을 시행할 것이다. 수술 후 처음으로 bed side에서 일어나는 시간 및 additional analgesics consumption에 대해 시간이 기록될 것이고 퇴원시까지 환자 상태에 대한 주기적인 모니터링을 수행할 것이다. 수술 후 합병증이 없는 상태에서 수술 7일차에 퇴원 및 수술 후 4주, 12주에 정기 외래 추시하며 증상 평가를 시행할 예정이다.

**<** **Postoperative basal analgesia>**

실험군과 대조군 모두에서 수술 후 pain control을 위해 다양한 루트를 통하여 pain management를 시행할 예정이다..

|  | postop 1day | Postop 2day - discharge |
| --- | --- | --- |
| Fluid | 1. Nefopam 60mg + 0.9% Normal saline  2.Oxycodone 10mg+0.9% Normal saline | 1. Nefopam 60mg + 0.9% Normal saline |
| PO medication | Traspen tab(Tramadol 37.5mg, Acetaminophen 325mg) 1T tid | Traspen tab(Tramadol 37.5mg, Acetaminophen 325mg) 1T tid |

**<Rescue analgesia>**

수술 후 VAS 4점 이상의 통증을 호소할 시 Tramadol 50mg IM(tramadol hydroxychloride), VAS 6점 이상의 통증을 호소할 시 Pethidine 25mg IM(pethidine hydroxychloride)으로 투약할 예정이다. 수술 후 통증 평가의 한 척도로 사용될 예정이며 퇴원 시까지의 투약 시간 및 횟수를 기록할 것이다.

**<결과 평가 방법>**

시험단계: 4상 / 시험기간: -

- 경추척수증, 혹은 경추신경근증으로 진단받고 후궁성형술 시행하는 환자 중 수술 전후 통증 변화 및 치료 효과, 예후를 파악한다. 주 결과변수는Visual analog scale(VAS) score로 확인한다.

1. Visual analog scale(VAS) score
2. Opioid consumption
3. Rescue analgesic consumption
4. Adverse effects
5. JOA(Japanese Orthopaedic Association), NDI(Neck disability index) score


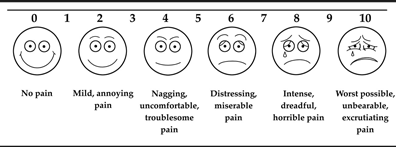


<Visual analog scale, VAS>


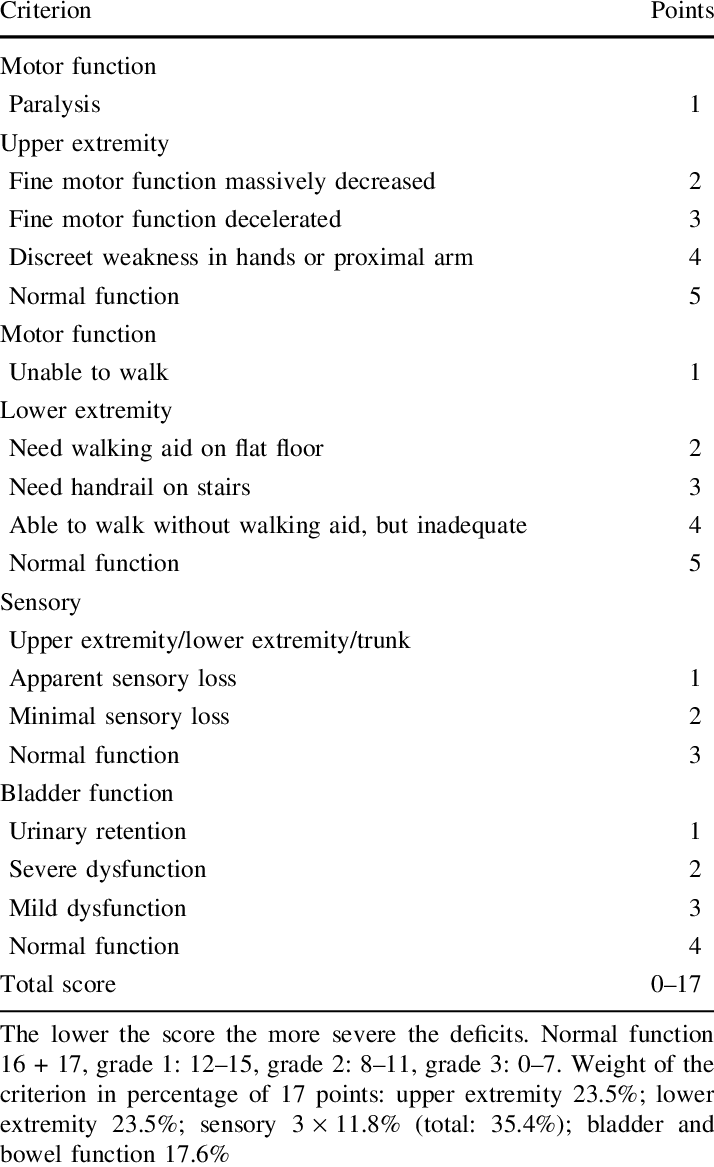


< Japanese Orthopaedic Association(JOA) score>


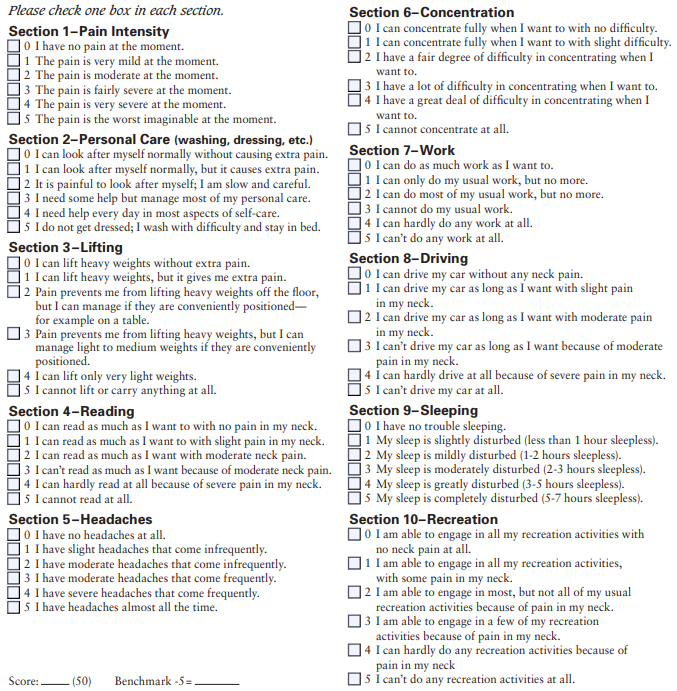


<NDI(Neck disability index) score>

입원 기간 중(~postop 7days)

1. 시험대상자를 참여시키기 전에 본 임상시험의 과정을 설명하고, 서면 동의서를 받는다
2. 시험대상자에게 동의를 받은 순서대로 스크리닝 번호를 부여한다
3. 시험대상자의 인구학적 정보(성별, 생년월일, 연령), 주호소증상(CC), 흡연여부, 병력(당뇨, etc), steroid 복용 여부를 조사한다
4. 활력징후(혈압, 맥박) 측정, BMD, 신체검진(Spurling test, shoulder abduction relief, tandem gait, grip & release) 및 신체계측(BMI)을 실시한다
5. 실험실 검사(혈액학적, 혈액화학적, 소변검사)를 실시한다
6. 수술 후 1시간 및 6, 12, 24, 36, 48, 72시간, 7일, 1개월, 3개월에 Visual analogue scale score (VAS score)
7. IV-PCA 총 사용량 기록하여 opioid consumption을 기록한다.
8. 수술 후 PCA를 제외한 추가적인 rescue analgesia consumption을 측정한다.
9. 구역, 구토 등 부작용 발생 시 기록

10) 다음 방문일(수술 후 4주째)을 지정한다

2차 방문 (visit 2, 4weeks)

이 방문에서 이루어져야 하는 평가는 다음과 같다.

1. 내원 시 VAS 측정
2. NDI, JOA score 측정

3차 방문 (visit 3, 12weeks)

이 방문에서 이루어져야 하는 평가는 다음과 같다.

1. 내원 시 VAS 측정
2. NDI, JOA score 측정

**<표본 크기>**

Sample size는 power analysis(standard deviation 2σ, a=0.05, 2-sided; power=80%) 으로 설정하였고, 기존 pliot study에서 실험군의 mean VAS 3 / 대조군의 mean VAS 1로 설정 후 구한 sample size는 25/25, total 50으로 탈락되는 실험자의 비율을 20% 으로 가정했을 때, total 64의 표본수가 필요하다.

- Chaiwat K et al, Spine, 2020의 연구를 pilot study로 하여 수술 후 24시간 이후 VAS score 참고하여 작성함.
- 통계방법은 SPSS software (version 25.0; IBM Corp., Armonk, NY, USA)을 이용하여 다음과 같은 통계학적 기술을 사용할 예정이다. unpaired t-test, Wilcoxon rank-sum test, chi-square test, Fisher’s exact test.

**<기존의 표준치료방법>**

- 본 연구에서 사용되는 설문이나 이학적 검사방법은 현재 본원에서 경추부 신경근증 혹은 척수증으로 치료받고 있는 환자를 대상으로 하는 검사방법으로 본 연구에서 시행하는 추가적인 연구방법은 없음.

7. 본 연구의 유용성

척추 수술은 수술 후 통증 관리에 어려움이 많은 분야로 수술 후 환자의 만족도를 높이고 술 후 재활을 순조롭게 진행하여 환자를 일상으로 빠르게 복귀할 수 있도록 하기 위해서는 수술 후 통증 조절이 무엇보다 중요하다.(27, 28) Mullen et al(29)에 의해 lumbar spine 수술에서 local anesthetic infiltration의 효과가 처음 알려진 이후 이와 같은 방식의 통증 조절은 지금까지 널리 이용되고 있다. 2012년 Kjergaard et al(30)은 그 효과에 대해 명확히 하고자 systemic review를 하였으나 heterogeneity of inclusion criteria, choice of agent and operative technique and low power of the studies 등으로 인해 의미있는 분석을 하기 어려워 의미있는 결론을 도출해내지 못하였다. 이후 2017년 Perera et al(23) 은 Lumbar spine 수술 후 intramuscular local analgesics injection의 효과에 대해 시행한 11개의 prospective RCT를 분석한 meta-analysis에서 수술 후 opioid 요구를 줄이고 첫번째 진통제 요구 시간의 연장, 술 후 1시간째의 vas score 감소에 효과적이었다고 보고하였다. 이와 같이 lumbar spine에 대해서는 local analgesic infiltration의 효과에 대한 보고가 있으나 cervical spine 수술에서의 사용 및 그 효용성을 분석한 연구 결과는 부족한 실정이다. 따라서 본 연구는 Cervical laminoplasty 수술 후 multimodal cocktail injection의 효과 및 안전성에 대해 분석한 최초의 prospective randomized controlled trail이라는 측면에서 그 의미가 있으며 본 연구를 통해 척추 수술 전반에 있어 multimodal cocktail analgesic injection이 유용할 가능성을 제공할 수 있을 것으로 기대한다.

8. 중지/탈락 기준

시험대상자는 본인의 요청에 의해 언제든지 시험을 조기 중단하거나 안전,

행위 또는 행정상의 이유로 연구자의 재량에 의해 언제든지 중도 탈락할 수 있다. 시험자는 중도탈락 사유에 대하여 질문하고, 마지막 방문을 하도록 요청하고, 해결되지 않은 이상반응이 있는 경우 시험대상자를 관찰하도록 최대한 노력하여야 한다.

임상시험이 조기 중단될 수 있는 경우는 다음과 같다.

1. 시험대상자가 임상시험 참여 동의를 철회한 경우
2. 선정/제외기준에 위배된 경우
3. 중대한 임상시험계획서 위반인 경우
4. 시험대상자에게 임상시험용 의약품을 투여하는 데 문제가 있는 경우
5. 이상반응으로 인하여 시험지속이 곤란한 경우
6. 안정선/유효성 평가에 영향을 미치는 병용약물의 투여 또는 해당 약물의 투여가 필요하다고 판단한 경우
7. 시험대상자의 추적관찰이 불가능한 경우
8. 시험자가 시험대상자의 시험지속이 부적절하다고 판단한 경우
9. 여성 시험대상자가 임상시험용 의약품 투여 중 임신 사실을 알게 된 경우

9. 효과 평가기준, 평가방법 및 해석방법 (통계분석방법)

1) 양 군의 수술 후 pain에 대해 VAS score 통계적으로 비교한다.

2) 추가적으로 Visual analog scale(VAS) score, Opioid consumption, rescue analgesic consumption, adverse effects, NDI, JOA score에 대해 통계적으로 비교한다.

10. 본 연구로 인하여 피험자에게 예상되는 위험

발생 가능한 부작용 ／ 이상반응 :

(1)로피바(Ropivacaine)

1)심혈관계: 고혈압, 빈맥, 흉통,저혈압, 서맥

2) 중추신경계: 발열, 두통, 어지럼증, 오한, 불안, 현기증

3) 피부계: 소양증

4) 대사내분비계: 저칼륨혈증

5) 비뇨생식기계: 요정체, 요로감염

6) 혈액학적: 빈혈

7) 근신경계 & 골격근계: 감각이상, 감각저하, 경직, 입주위감각이상

8) 신장: 소변감소증

9) 호흡기계: 호흡곤란

10) 기타: 몸의 떨림

(2)탐세톤(Triamcinolone)

1) 피부: 가려움, 알레르기성 접촉 피부염, 건조, 모낭염, 피부염, 홍반, 건성 피부, 여드름 모양, 짓무름, 피부 위축, 위축선, 한진, 구강 점막 위축

2) 국소: 작열감, 자극감

(3)에피네프린(Epinephrine)

1) 심혈관계: 협심증, 심부정맥, 흉통, 홍조, 고혈압, 심근 산소 소모량 증가, 창백, 심계항진, 돌연사, 빈맥(주사), 혈관수축, 심실전위

2) 중추신경계: 불안, 어지럼증, 두통, 불면증, 현기증, 신경과민, 안절부절

3) 위장관계: 목건조, 오심, 구토, 구강건조증

4) 비뇨생식기계: 방광 유출 폐색 환자에서 급성 뇨저류

5) 근골격계: 진전, 허약

6) 안과: 알러지성 안검반응, 작열감, 눈의 통증, 눈자극감, 협우각 녹내장 악화, 일과성 자통

7) 신장: 신장 또는 내장혈류 감소

8) 호흡기: 호흡곤란, 천명

9) 기타: 발한증가

(4)케토신(Ketorolac)

1) 소화기계: 구역, 소화불량, 위장관통증, 설사, 때때로 변비, 복부팽만감, 구토, 구내염, 드물게 소화성 궤양, 위장관출혈, 천공, 혈변, 직장출혈, 위염, 트림, 식욕부진, 식욕증진, 췌장염, 토혈, 식도염, 흑색변, 입건조, 대장염 및 크론병 악화 등이 나타날 수 있다.

2) 전신: 때때로 부종, 드물게 아나필락시스, 기관지경련, 후두부종, 혀부종, 저혈압, 조홍, 발진과 같은 과민반응, 체중증가, 발열, 감염, 무력증이 나타날 수 있다.

3) 심혈관계: 고혈압, 드물게 홍조, 심계항진, 창백, 저혈압, 실신, 서맥, 가슴통증, 심부전이 나타날 수 있다.

4) 피부: 발적, 때때로 가려움, 드물게 독성표피괴사용해(리엘증후군), 피부점막안증후군(스티븐스-존슨 증후군), 박탈성 피부염, 구진성 발적, 두드러기, 혈관부종, 발한이 나타날 수 있다.

5) 혈액 및 림프계: 자반병, 드물게 수술부위 출혈, 혈소판감소증, 코피, 빈혈, 백혈구감소증, 호산구증다증, 혈종이 나타날 수 있다.

6) 신경계: 두통, 발한, 때때로 졸음, 어지러움, 드물게 경련, 진전, 환각, 비정상적인 꿈, 이상황홀감, 추체외로 증후군, 지각이상, 우울, 불면, 신경과민, 구갈, 심한 갈증, 비정상적 생각, 집중력장애, 과운동증, 혼미, 근육통, 무균성 수막염(특히 전신홍반루프스(SLE) 및 혼합결합조직병(MCTD)과 같은 자가면역질환을 앓고 있는 환자), 불안, 정신병적 반응이 나타날 수 있다.

7) 간장: 드물게 간염, 간부전, 담즙울체성 황달, 간기능 검사치의 이상이 나타날 수 있으며, 이러한 증상이 나타나는 경우에는 투여를 즉시 중지한다.

8) 호흡기계: 드물게 호흡곤란, 천식, 폐부종, 비염, 기침이 나타날 수 있다.

9) 비뇨기계: 드물게 급성신부전, 측복부통증(혈뇨 및 고질소혈증을 동반하기도 함), 핍뇨, 신염, 다뇨, 빈뇨, 요저류, 용혈성 요독증, 고칼륨혈증, 저나트륨혈증, 신증후군, 혈청 요소 및 크레아티닌수치 상승이 나타날 수 있다.

10) 기타: 때때로 주사부위통증, 미각이상, 시각이상, 시야흐림, 시신경염, 이명, 청각상실, 여성에서 불임, 출혈시간연장이 나타날 수 있다.

(5)제티암(Cefotiam)

1) 쇽 : 드물게 쇽을 일으킬 수 있으므로 충분히 관찰하고 불쾌감, 구내이상감, 천명, 어지러움, 변의, 이명, 발한 등이 나타나는 경우에는 투여를 중지하고 적절한 처치를 한다.

2) 과민반응 : 때때로 발진, 두드러기, 홍반, 가려움, 발열, 림프절 종창, 관절통 등이 나타나는 경우에는 투여를 중지하고 적절한 처치를 한다.

3) 피부 : 드물게 스티븐스-존슨증후군, 독성표피괴사용해가 나타날 수 있으므로 충분히 관찰하고 이상이 인정되는 경우에는 투여를 중지하고 적절한 처치를 한다.

4) 혈액계 : 때때로 빈혈, 과립구 감소, 적혈구 감소, 호산구 증가, 혈소판 감소 드물게 범혈구 감소, 용혈성 빈혈이 나타날 수 있으므로, 이상이 인정되는 경우에는 투여를 중지하고, 적절한 처치를 한다.

5) 간장 : 때때로 AST, ALT, ALP의 상승 드물게 황달, LDH, γ-GTP상승 등이 나타날 수 있다.

6) 신장 : 드물게 급성 신부전 등의 중증의 신장애가 나타날 수 있으므로 정기적으로 검사를 실시하는 등 충분히 관찰하고 이상이 인정되는 경우에는 투여를 중지하고 적절한 처치를 한다.

7) 소화기계 : 드물게 위막성대장염 등의 혈변을 수반하는 중증의 대장염이 나타날 수 있다. 복통, 빈번한 설사가 나타나는 경우에는 즉시 투여를 중지하는 등 적절한 처치를 한다. 또한 때때로 구역, 설사 또한 드물게 구토, 식욕부진, 복통 등이 나타날 수 있다.

8) 호흡기계 : 드물게 발열, 기침, 호흡곤란, 흉부 X선 이상, 호산구 증가 등을 수반하는 간질성 폐렴, 호산구성 폐침윤 등이 나타날 수 있으므로 이러한 증상이 나타나는 경우에는 투여를 중지하고 코르티코이드 투여 등 적절한 처치를 한다.

9) 중추신경계 : 신부전 환자에 대량 투여 시 경련 등을 일으킬 수 있다.

10) 균교대증 : 드물게 구내염, 칸디다증이 나타날 수 있다.

11) 비타민 결핍증 : 드물게 비타민 K 결핍증상(저프로트롬빈혈증, 출혈경향 등), 비타민 B군 결핍증상(설염, 구내염, 식욕부진, 신경염 등)이 나타날 수 있다.

12) 기타 : 드물게 어지러움, 두통, 권태감, 마비감이 나타날 수 있다

(6)모르핀(Morphine)

1) 의존성 : 계속 투여로 약물의존성이 생길 수 있으므로 충분히 관찰하고 신중히 투여한다. 계속 투여 중 투여량을 급격히 감소시키거나 투여를 중지할 때 하품, 재채기, 눈물흘림, 땀흘림, 구역, 구토, 설사, 복통, 동공 확대, 두통, 불면, 불안, 헛소리, 경련, 떨림, 전신의 근육과 관절의 통증, 호흡촉박, 심계항진 등의 증상이 나타나면 1일 투여량을 서서히 감량하면서 환자의 상태를 신중히 관찰한다.

2) 호흡억제 : 호흡곤란, 느린호흡, 불규칙호흡, 무호흡 등의 호흡억제 증상이 나타날 수 있으므로, 충분히 관찰하고 증상이 나타나면 마약길항제(날록손 등) 투여, 호흡보조 등의 적절한 처치를 한다. 정맥내 투여 시 최대 중추신경계 효과가 30분 정도 지연되어 나타나기 때문에, 급속히 투여하면 과량투여가 될 수 있다. 경막외 또는 수막강내로 급속주사하면 이 약이 뇌 호흡중추에 직접 정맥 재분포하여 조기 호흡억제가 나타날 수 있는데, 이러한 호흡억제는 최대 24시간까지 지연발현될 수 있다. 수막강내 투여 시 경막외 투여보다 호흡억제가 더 빈번히 나타났으나, 대부분 권장용량보다 훨씬 고용량을 투여한 경우였다.

3) 두부손상과 두개내압 상승 : 이 약의 호흡억제 작용으로 인한 2차적인 뇌척수액 압력 증가는 두부손상, 다른 두개내 병변, 또는 이미 있던 두개내압 상승을 더욱 악화시킬 수 있다. 또한 마약성 진통제는 두부손상이 있는 환자의 임상적 경과를 불명확하게 할 수 있다.

4) 혈압저하 및 쇽 : 이 약을 포함한 마약성 진통제는 혈량부족 또는 페노티아진계 약물, 전신마취제 등과의 병용으로 인해 혈압유지능력이 떨어져 있는 환자에게 중증 저혈압 및 쇽을 일으킬 수 있다. 이 약에 의해 혈관확장이 유발되어 심박출량과 혈압을 더욱 감소시킬 수 있으므로 순환기계 쇽이 있는 환자에게는 주의하여 투여한다.

5) 1일 20 mg을 초과하는 양을 수막강내 투여한 환자에게서 하지의 간대성근연축이 보고되었다.

6) 순환기계 : 저용량을 정맥내 투여한 경우에는 심혈관계의 안정성에 거의 영향을 미치지 않으나, 고용량 투여 시 순환 카테콜아민의 증가로 인한 교감신경계 과활성화 및 흥분으로 인한 경련이 나타날 수 있다.

7) 중추신경계 : 고용량 정맥내 투여 시 중추신경계를 흥분시켜 경련이 일어날 수 있다.

8) 정신신경계 : 불쾌감, 불안, 중독성정신병, 이상황홀감, 착란, 두통, 이질통, 통각과민, 다한증이 나타날 수 있다.

9) 소화기계 : 구역, 구토가 나타날 수 있는데, 저용량(0.2 mg)의 날록손 투여로 증상을 경감시킬 수 있다. 변비, 담도경련, 구강건조가 나타날 수 있다.

10) 비뇨기계 : 약 90 %의 남성환자에서 경막외나 수막강내 단회투여 후 10～20시간 동안 요저류가 지속되었으나, 여성에서는 그 발생률이 낮았다. 때때로 요카테터 삽입이 필요할 수도 있으며, 카테터 삽입 시 이로 인한 이상반응(예: 패혈증)에 주의한다. 저용량(0.2 mg)의 날록손 투여로 증상을 경감시킬 수 있다. 핍뇨가 나타날 수 있다.

11) 피부 : 가려움이 나타날 수 있는데, 이는 투여량과 관련이 있으며 투여부위와는 무관하다. 저용량(0.2 mg)의 날록손 투여로 증상을 경감시킬 수 있다.

12) 과민반응 : 두드러기, 국소조직자극반응이 나타날 수 있다.

13) 기타 : 기침반사억제, 남녀의 성욕감소, 월경불순, 무월경, 체온조절 장애가 나타날 수 있다.

14) 위장관계 : 장폐쇄

11. 부작용을 포함한 안전성의 평가 및 보고방법

양 군간 부작용에 대해 기록 및 평가, 보고한다.

12. 임상시험용의약품의 정보 및 관리

1. 임상시험용 의약품

(1) 로피바 주사(ROPIVA Inj. Hanlim Pharm Co., Ltd)

① 성분 및 함량: ropivacaine hydrochloride hydrate 7.9㎎/㎖

② 제형 및 성상: 주사제

③ 보관 방법: 밀봉용기, 실온보관(1~30℃)

④ 제조원: 한림제약

⑤ 용법/용량: 1일 3회, 1회 1정 경구 투여

(2) 탐세톤 주사(TAMCETON 40 INJ**.** Hanall Biopharma)

① 성분 및 함량: Triamcinolone Acetonide 40mg/mL

② 제형 및 성상: 주사제

③ 보관 방법: 차광밀봉용기, 실온(1~30℃)보관

④ 제조원: 한올바이오파마

(3) 대한 에피네프린 주사액(EPINEPHRINE DAIHAN INJ. Daihan Pharm Co., Ltd)

① 성분 및 함량: Epinephrine 1mg/mL

② 제형 및 성상: 주사제

③ 보관 방법: 차광밀봉용기, 25℃이하 보관, 냉장보관하지 말 것.

④ 제조원: 대한약품공업

(4) 케토신 주(KETOCIN INJ 30mg/ml. Myungmoon Pharm. Co., Ltd)

① 성분 및 함량: Ketorolac tromethamine 30㎎/㎖

② 제형 및 성상: 주사제

③ 보관 방법: 차광밀봉용기, 실온(1-30℃)보관

④ 제조원: 명문제약

(5) 제티암 주(JETIAM INJ 1g. Samjin Pharm)

① 성분 및 함량: Cefotiam Hydrochloride-Dried Sodium Carbonate 1.242g

② 제형 및 성상: 주사제

③ 보관 방법: 밀봉용기, 실온(1~30℃)보관

④ 제조원: 삼진제약

(6) 비씨 모르핀황산염수화물 주사(BC MORPHINE SULFATE INJ 1mg/mL)

① 성분 및 함량: Morphine Sulfate hydrate 1mg/mL

② 제형 및 성상: 주사제

③ 보관 방법: 차광밀봉용기, 실온보관(1-30℃)

④ 제조원: 비씨월드제약

 =======================================================

13. 피험자동의서 양식

수술 후 통증 평가를 위한 연구에 있어 환자 동의서가 필요함.

14. 피해자 보상에 대한 규약

별첨

15. 증례기록서 양식

별첨

16. 임상시험후 피험자의 진료 및 치료기준

일반적으로 경추 척수증 및 신경근증 외래 환자에서 시행하는 방식으로 외래 방문 시 증상 변화에 대한 설문지 작성을 시행하게 됨.

17. 피험자의 안전보호에 관한 대책

피험자의 정보는 어디에도 공개되지 않으며, 피험자는 성별, 나이, 만을 기록하고 순서상으로 코드화하여 결과만을 기록하므로 사생활보호와 비밀보장에 전혀 문제가 없을 것으로 사료됨. 시험자는 Helsinki 선언에 입각하여 대상자의 권리와 복지를 염두에 두고 임상시험을 실시하여야 하며, 본 임상시험에 참여하는 시험자들은 의약품임상시험관리기준, 임상시험계획서 등에 대하여 숙지하고 임상시험을 실시하여야 한다. 시험자는 각 대상자에게 충분한 시간을 할애하여 면담 및 검사를 통하여 대상자 적합여부 및 이상반응 발생 여부에 대하여 철저히 평가한다. 공동연구자는 연구책임자에게 주기적으로 이상반응, 시험진행, 상황, 결과 등에 대하여 보고하며, 연구 담당자는 주기적으로 임상시험 진행 상황에 대하여 관리한다.

18. 자료 기록, 보관 방법

임상연구와 관련된 분서는 연구 종료 후 본원의 문서보관 책임자에게 인계하여 3년간 보관하게 됨.

19. Audit 및 Monitoring 계획

피험자의 권리와 복지 보호, 임상시험관련 자료가 근거 문서와 대조하여 정확하고 완전하며, 검증 가능한지 여부 확인, 임상시험계획서 준수 여부 및 관련법규를 준수하여 임상시험을 진행하였는지를 확인하기 위해 시험책임자는 임상시험심사위원회에 점검(audit) 및 모니터링(monitoring)을 신청하거나, 임상시험심사위원회 점검시 적극 협조한다.

**References**

1. Sakai Y, Matsuyama Y, Inoue K, Ishiguro N. Postoperative instability after laminoplasty for cervical myelopathy with spondylolisthesis. J Spinal Disord Tech. 2005;18(1):1-5.

2. Kimura A, Seichi A, Inoue H, Hoshino Y. Long-term results of double-door laminoplasty using hydroxyapatite spacers in patients with compressive cervical myelopathy. Eur Spine J. 2011;20(9):1560-6.

3. Cho SK, Kim JS, Overley SC, Merrill RK. Cervical Laminoplasty: Indications, Surgical Considerations, and Clinical Outcomes. J Am Acad Orthop Surg. 2018;26(7):e142-e52.

4. Weinberg DS, Rhee JM. Cervical laminoplasty: indication, technique, complications. J Spine Surg. 2020;6(1):290-301.

5. Trousdale RT, McGrory BJ, Berry DJ, Becker MW, Harmsen WS. Patients' concerns prior to undergoing total hip and total knee arthroplasty. Mayo Clin Proc. 1999;74(10):978-82.

6. Park KK, Shin KS, Chang CB, Kim SJ, Kim TK. Functional disabilities and issues of concern in female Asian patients before TKA. Clin Orthop Relat Res. 2007;461:143-52.

7. Capdevila X, Barthelet Y, Biboulet P, Ryckwaert Y, Rubenovitch J, d'Athis F. Effects of perioperative analgesic technique on the surgical outcome and duration of rehabilitation after major knee surgery. Anesthesiology. 1999;91(1):8-15.

8. Morrison SR, Magaziner J, McLaughlin MA, Orosz G, Silberzweig SB, Koval KJ, et al. The impact of post-operative pain on outcomes following hip fracture. Pain. 2003;103(3):303-11.

9. Deng Z, Li Y, Storm GR, Kotian RN, Sun X, Lei G, et al. The efficiency and safety of steroid addition to multimodal cocktail periarticular injection in knee joint arthroplasty: a meta-analysis of randomized controlled trials. Sci Rep. 2019;9(1):7031.

10. Chelly JE, Ben-David B, Williams BA, Kentor ML. Anesthesia and postoperative analgesia: outcomes following orthopedic surgery. Orthopedics. 2003;26(8 Suppl):s865-71.

11. Kehlet H, Dahl JB. The value of "multimodal" or "balanced analgesia" in postoperative pain treatment. Anesth Analg. 1993;77(5):1048-56.

12. Burroughs TE, Davies AR, Cira JC, Dunagan WC. Understanding patient willingness to recommend and return: a strategy for prioritizing improvement opportunities. Jt Comm J Qual Improv. 1999;25(6):271-87.

13. Brokelman RB, van Loon CJ, Rijnberg WJ. Patient versus surgeon satisfaction after total hip arthroplasty. J Bone Joint Surg Br. 2003;85(4):495-8.

14. Fisher CG, Belanger L, Gofton EG, Umedaly HS, Noonan VK, Abramson C, et al. Prospective randomized clinical trial comparing patient-controlled intravenous analgesia with patient-controlled epidural analgesia after lumbar spinal fusion. Spine (Phila Pa 1976). 2003;28(8):739-43.

15. Yukawa Y, Kato F, Ito K, Terashima T, Horie Y. A prospective randomized study of preemptive analgesia for postoperative pain in the patients undergoing posterior lumbar interbody fusion: continuous subcutaneous morphine, continuous epidural morphine, and diclofenac sodium. Spine (Phila Pa 1976). 2005;30(21):2357-61.

16. Jirarattanaphochai K, Jung S. Nonsteroidal antiinflammatory drugs for postoperative pain management after lumbar spine surgery: a meta-analysis of randomized controlled trials. J Neurosurg Spine. 2008;9(1):22-31.

17. Wheeler M, Oderda GM, Ashburn MA, Lipman AG. Adverse events associated with postoperative opioid analgesia: a systematic review. J Pain. 2002;3(3):159-80.

18. Block BM, Liu SS, Rowlingson AJ, Cowan AR, Cowan JA, Jr., Wu CL. Efficacy of postoperative epidural analgesia: a meta-analysis. JAMA. 2003;290(18):2455-63.

19. Kurosaka K, Tsukada S, Seino D, Morooka T, Nakayama H, Yoshiya S. Local Infiltration Analgesia Versus Continuous Femoral Nerve Block in Pain Relief After Total Knee Arthroplasty: A Randomized Controlled Trial. J Arthroplasty. 2016;31(4):913-7.

20. Li D, Tan Z, Kang P, Shen B, Pei F. Effects of multi-site infiltration analgesia on pain management and early rehabilitation compared with femoral nerve or adductor canal block for patients undergoing total knee arthroplasty: a prospective randomized controlled trial. Int Orthop. 2017;41(1):75-83.

21. Nakai T, Nakamura T, Nakai T, Onishi A, Hashimoto K. A study of the usefulness of a periarticular multimodal drug cocktail injection for pain management after total hip arthroplasty. J Orthop. 2013;10(1):5-7.

22. Salwan A, Pisulkar GL, Taywade S, Awasthi AA, Saoji A, Jadawala VH, et al. A Review on the Efficacy of Extraosseous Local Infiltration of Multimodal Drug Cocktail for Pain Management After Total Knee or Hip Arthroplasty. Cureus. 2022;14(10):e30451.

23. Perera AP, Chari A, Kostusiak M, Khan AA, Luoma AM, Casey ATH. Intramuscular Local Anesthetic Infiltration at Closure for Postoperative Analgesia in Lumbar Spine Surgery: A Systematic Review and Meta-Analysis. Spine (Phila Pa 1976). 2017;42(14):1088-95.

24. Kraiwattanapong C, Arnuntasupakul V, Kantawan R, Woratanarat P, Keorochana G, Langsanam N. Effect of Multimodal Drugs Infiltration on Postoperative Pain in Split Laminectomy of Lumbar Spine: A Randomized Controlled Trial. Spine (Phila Pa 1976). 2020;45(24):1687-95.

25. Elder JB, Hoh DJ, Liu CY, Wang MY. Postoperative continuous paravertebral anesthetic infusion for pain control in posterior cervical spine surgery: a case-control study. Neurosurgery. 2010;66(3 Suppl Operative):99-106; discussion -7.

26. Southerland WA, Gillis J, Urits I, Kaye AD, Eskander J. Intraoperative Administration of Dexmedetomidine and Dexamethasone in Local Anesthetic Infiltration to Improve Postoperative Pain Control After Posterior Cervical Fusion. Cureus. 2021;13(4):e14699.

27. Bianconi M, Ferraro L, Ricci R, Zanoli G, Antonelli T, Giulia B, et al. The pharmacokinetics and efficacy of ropivacaine continuous wound instillation after spine fusion surgery. Anesth Analg. 2004;98(1):166-72.

28. Bajwa SJ, Haldar R. Pain management following spinal surgeries: An appraisal of the available options. J Craniovertebr Junction Spine. 2015;6(3):105-10.

29. Mullen JB, Cook WA, Jr. Reduction of postoperative lumbar hemilaminectomy pain with Marcaine. Technical note. J Neurosurg. 1979;51(1):126-7.

30. Kjaergaard M, Moiniche S, Olsen KS. Wound infiltration with local anesthetics for post-operative pain relief in lumbar spine surgery: a systematic review. Acta Anaesthesiol Scand. 2012;56(3):282-90.
