# Supplementary material for: The effects of multimodal cocktail analgesic local injection in postoperative pain control after laminoplasty: A study protocol of a prospective randomized controlled trial
Source: PLoS One. 2025 Jun 13;20(6):e0324791. doi: 10.1371/journal.pone.0324791 (PMC12165372; doi:10.1371/journal.pone.0324791)
Supplement: S3 File — (DOCX) [file pone.0324791.s003.docx]

【Study protocol】

**1. Study Title**

A prospective study on the comparison of postoperative pain according to the use of cocktail therapy in laminoplasty

**2. Name and Address of the Research Institution**

Department of Orthopaedic Surgery, Kyung-Hee University Medical Center, Kyung-Hee University School of Medicine, 23, Kyungheedae-ro, Dongdaemun-gu Seoul, Republic of Korea 130-872

TEL: 82-2-958-8346

E-mail: futurespine@gmail.com

**3. Names and Positions of Principal Investigator, Co-Investigator, and Research Coordinator**

Principal Investigator and Co-Investigator: Kang Kyung-Chung, MD (Professor)

Co-Investigator: Lee Won-Young, MD (Attending Physician)

Research Coordinator: Park Min-Jeong (CRC)

**4. Purpose and Background of the Research**

Laminoplasty is widely used in the surgical treatment of cervical myelopathy due to its advantages in preserving neck motion and being relatively safe. However, the pain experienced by patients after surgery is considerable, involving dissection of the posterior neck muscles and bone procedures. Patients undergoing surgery are often more concerned about the postoperative pain than the surgery itself, leading to cancellations or postponements. Poorly controlled postoperative pain can negatively impact rehabilitation, leading to extended hospital stays, increased readmission rates, and higher treatment costs. In particular, inadequate pain control can reduce patient satisfaction and become a significant factor in the patient's decision for additional treatments. Therefore, effective pain control after surgery is crucial for maintaining patient satisfaction and facilitating successful rehabilitation.

Various options exist for postoperative pain control, but individual drugs may have side effects that limit their use. The concept of multimodal analgesia, introduced by Kehlet and Dahl, involves maximizing the efficiency of various analgesics by using them in appropriate doses and diversifying the routes of administration to achieve maximum pain relief with minimal side effects. Applying this concept to surgery involves attempting pain control through localized drug administration to the surgical site. While this approach is widely used in various surgical fields, especially in orthopedic surgery and joint replacement surgeries, studies on the effects of multimodal cocktail analgesic local injection in cervical surgery are limited. This study aims to evaluate the effects of cocktail therapy on postoperative pain and its utility in patients undergoing laminoplasty for cervical myelopathy or cervical radiculopathy.

**Objective**: This study aims to evaluate the effects of cocktail therapy on postoperative pain and its utility in patients undergoing laminoplasty for cervical myelopathy or cervical radiculopathy.

**5. Criteria for Participant Selection**

**[Inclusion criteria]**

- Patients with cervical myelopathy or cervical radiculopathy planning to undergo laminoplasty at the hospital
- Patients with recorded preoperative upper limb pain (VAS), neck pain (VAS), JOA, and NDI scores
- Adults aged 20 to under 80 who can express their pain or functional impairment
- Patients who understand the study procedures and can comply with them

**[Exclusion criteria]**

- Patients with a history of surgical treatment in the cervical region
- Adolescents under 20 and pregnant women
- Patients with hypersensitivity reactions to the mixed drugs used in cocktail therapy (Morphine 5mg, ropiva 150mg, tamceton 40mg, epinephrine 1mg, ketocin 60mg, jetiam 1g)
- Patients lacking medical decision-making capacity or communication ability

**6. Methods**

**<Research Method>**

A single-center, prospective, randomized controlled trial will be conducted at the hospital. Patients diagnosed with cervical myelopathy or radiculopathy who undergo laminoplasty performed by a skilled single operator will be divided into groups based on whether intraoperative cocktail injection is performed.

**<Participants>**

Patients requiring laminoplasty for cervical myelopathy or radiculopathy will be selected after sufficient pre-explanation and obtaining informed consent from the researchers.

**Trial phase**: Phase 4 / Trial period: From IRB approval to September 30, 2025

**<Randomization>**

Random allocation will be conducted according to the block randomization method with a planned block size of 4. Randomized trial participants will be evaluated for efficacy and safety through hospitalization and outpatient visits four weeks after surgery.


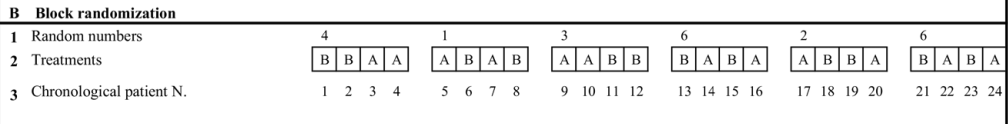


**<Interventions>**

All patients will be randomized into either the cocktail group or the control group. Patients in the cocktail group will receive multimodal cocktail analgesic injection into the perimuscular area just before wound closure, while patients in the control group will receive an equivalent volume of saline in the same manner. All procedures will be performed by a skilled single surgeon, and all patients will undergo cervical laminoplasty.

**<Blinding>**

Cocktail analgesics or an equivalent amount of saline will be prepared independently in a sterile manner in the operating room. Both the investigator and the patient will be kept in a double-blind state regarding group assignment. Data will be collected by a blinded researcher regarding group allocation, and after the completion of the study, data analysis will be performed by a statistical expert who is blind to group assignment information.

**<Double-blinding method>**

In this clinical trial, a double-blind method will be implemented using a placebo with the same formulation and appearance to ensure indistinguishability during the administration period. This will prevent both the investigator and the trial subjects from discerning to which group they belong. Maintaining blinding is crucial for the integrity of the trial, and group assignment information will be kept blinded to everyone, including the investigator, until the clinical trial database is locked and stored. It should be noted that in the process of evaluating and reporting Suspected Unexpected Serious Adverse Reactions (SUSAR), blinding may be lifted. Therefore, individuals other than the code manager (unblinding personnel) involved in evaluating and reporting SUSAR should not be involved in unblinding to maintain blinding. Randomization codes for the clinical trial drug will be managed, distributed, and returned by the code manager (unblinding personnel). Visually, the test drug and the placebo will have identical appearances, making them indistinguishable. As the investigational drug is administered while the patient is lying down, and it is handled by a separate administrator, patient blinding is possible.

- Additional Personnel for Blinding

Blinding Personnel: PI, Kang Kyung-Chung, CRC Park Min-Jeong

Unblinding Personnel: Sub-I, Lee Won-Young (IP management and administration)

**<Cocktail Analgesic Injection Group (Cocktail Group)>**

Cocktail Therapy Regimen : A total of 40ml injection, consisting of Morphine 5mg, ropiva 150mg, tamceton 40mg, epinephrine 1mg, ketocin 60mg, jetiam 1g, mixed with normal saline.

- Experimental Group: Injection of cocktail therapy into the deep fascia and muscular layer at wound closure
- Control Group: Injection of 40ml normal saline at wound closure

Both experimental and control groups will use IV-PCA for postoperative pain control. Postoperative pain control will be initiated with a fentanyl IV bolus of 50mcg immediately after surgery, followed by IV-PCA with a basal infusion of 0.3mcg/kg/h for 48 hours. A 15-minute lockout time setting will be applied, allowing bolus doses of 0.075mcg/kg. The infusion device will be removed after 48 hours, and the time and amount taken until the first use of IV-PCA will be compared.

**<Sterile Saline Injection Group (Control Group)>**

At wound closure, 40ml of normal saline will be injected into the deep fascia and muscular layer. Postoperative pain control will be performed in the same manner as the cocktail group.

**<Perioperative Management>**

The surgery will be performed under standardized general anesthesia. Fluid management during surgery will follow a standardized plan, including the use of balanced crystalloid and hydroxyethyl starch colloid. IV-PCA will be connected after surgery and removed 48 hours postoperatively. Postoperative care will include monitoring in the recovery room, evaluation of patient status according to standardized criteria, and transfer to the ward. The time taken for the first bedside mobilization after surgery and additional analgesics consumption will be recorded until discharge. Patients will be regularly monitored for the first 24 hours for patient status and recovery of lung function through deep breathing and coughing. If there are no complications, patients will be discharged on the 7th day after surgery and scheduled for regular outpatient visits at 4 and 12 weeks after surgery for symptom assessment.

**<Postoperative Basal Analgesia>**

Both the experimental and control groups will use various routes for pain management during the postoperative period.

|  | postop 1day | Postop 2day - discharge |
| --- | --- | --- |
| Fluid | 1. Nefopam 60mg + 0.9% Normal saline  2.Oxycodone 10mg+0.9% Normal saline | 1. Nefopam 60mg + 0.9% Normal saline |
| PO medication | Traspen tab(Tramadol 37.5mg, Acetaminophen 325mg) 1T tid | Traspen tab(Tramadol 37.5mg, Acetaminophen 325mg) 1T tid |

**<Rescue Analgesia>**

If a trial subject complains of pain with a VAS score of 4 or higher, Tramadol 50mg IM (tramadol hydrochloride) will be administered. If the VAS score is 6 or higher, Pethidine 25mg IM (pethidine hydrochloride) will be administered. These rescue analgesics will be used as needed, and the time and frequency of administration will be recorded until discharge.

**<Outcome measure>**

Trial Phase: Phase 4

Patients diagnosed with cervical myelopathy or cervical radiculopathy who undergo posterior cervical laminoplasty will be assessed for changes in pain, treatment effects, and prognosis before and after surgery. The primary outcome variable will be assessed using the Visual Analog Scale (VAS) score.

1. Visual Analog Scale (VAS) Score
2. Opioid Consumption
3. Rescue Analgesic Consumption
4. Adverse Effects
5. JOA (Japanese Orthopaedic Association) and NDI (Neck Disability Index) Scores


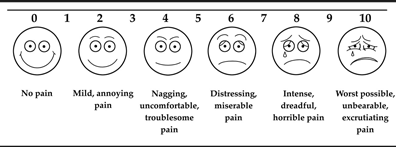


<Visual Analog Scale, VAS>


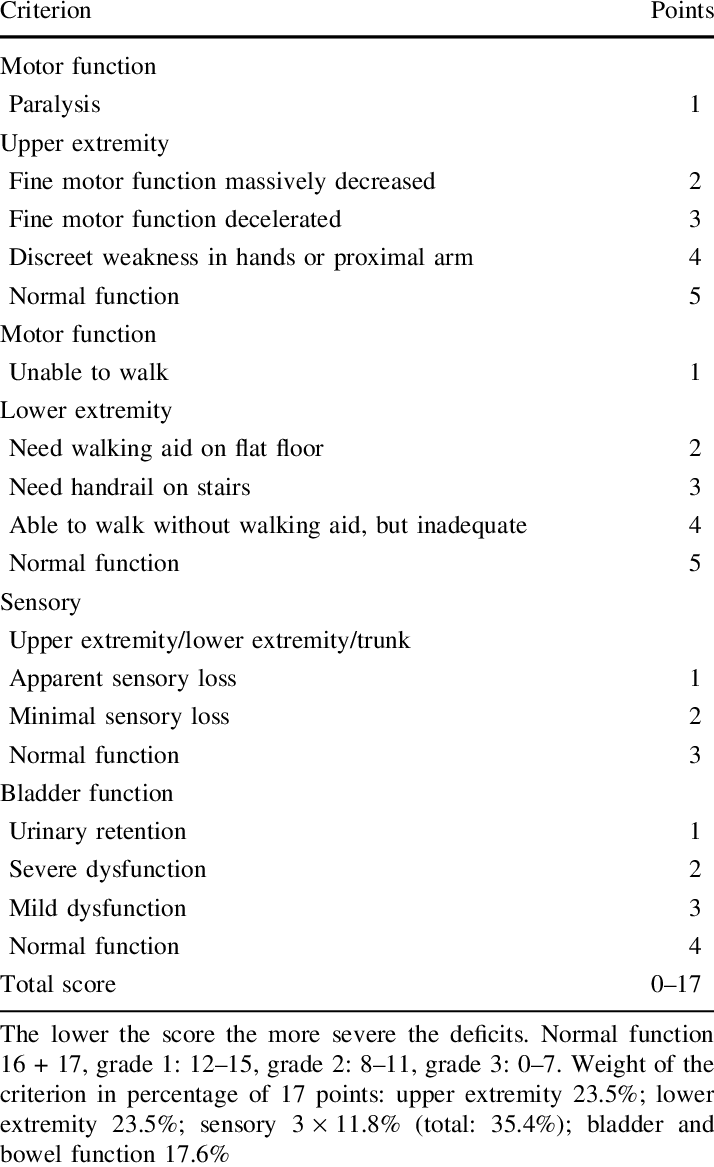


<Japanese Orthopaedic Association (JOA) Score>


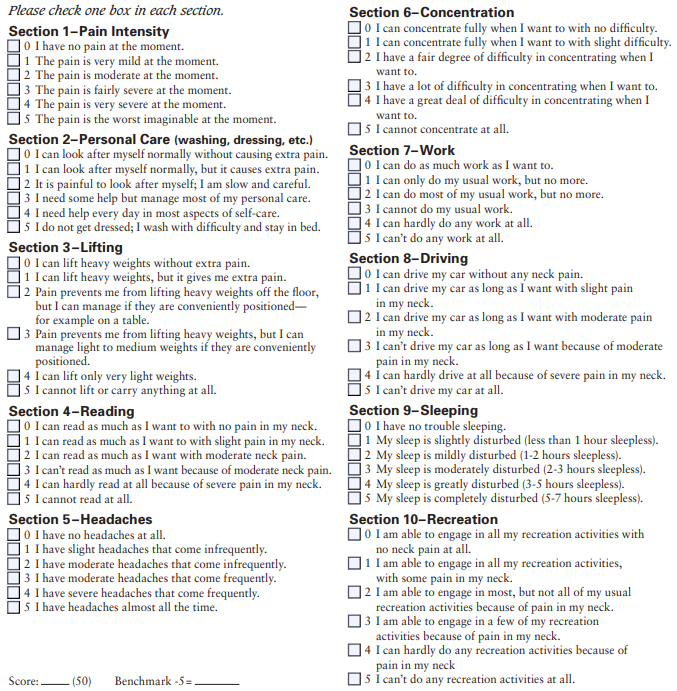


<Neck Disability Index (NDI) Score>

**During the hospitalization period (~postop 7 days):**

1. Explain the clinical trial process to potential subjects and obtain written consent before their participation.
2. Assign screening numbers to subjects in the order of obtaining consent.
3. Collect demographic information (gender, date of birth, age), chief complaints (CC), smoking status, medical history (diabetes, etc.), and steroid use for each subject.
4. Perform vital sign measurements (blood pressure, pulse), BMD, physical examinations (Spurling test, shoulder abduction relief, tandem gait, grip & release), and body measurements (BMI).
5. Conduct laboratory tests (hematological, biochemical, urinalysis).
6. Measure Visual Analog Scale scores (VAS score) at 1 hour, 6, 12, 24, 36, 48, 72 hours, 7 days, 1 month, and 3 months post-surgery.
7. Record total IV-PCA usage to document opioid consumption.
8. Measure additional rescue analgesia consumption excluding PCA after surgery.
9. Record any side effects such as nausea and vomiting.
10. Schedule the next visit (4 weeks post-surgery).

**Second Visit (Visit 2, 4 weeks):**

Evaluation at this visit includes:

1. Measurement of VAS score during the visit.
2. Measurement of NDI and JOA scores.

**Third Visit (Visit 3, 12 weeks):**

Evaluation at this visit includes:

1. Measurement of VAS score during the visit.
2. Measurement of NDI and JOA scores.

**<Sample Size>**

The sample size was determined through power analysis (standard deviation 2σ, α=0.05, 2-sided; power=80%). The sample size, based on the mean VAS of 3 for the experimental group and mean VAS of 1 for the control group from a previous pilot study, was calculated as 25/25, totaling 50. Assuming a dropout rate of 20%, a total sample size of 64 is required.

- Based on Chaiwat K et al, Spine, 2020, as a pilot study, referring to the VAS score 24 hours after surgery.
- Statistical methods using SPSS software (version 25.0; IBM Corp., Armonk, NY, USA) will include unpaired t-test, Wilcoxon rank-sum test, chi-square test, Fisher’s exact test.

**<Existing Standard Treatment Methods>**

- The surveys and diagnostic methods used in this study are procedures targeting patients at the clinic who are undergoing treatment for cervical myelopathy or cervical radiculopathy. No additional research methods are conducted in this study.

**7. Usefulness of the Study**

Spinal surgery is an area with significant challenges in postoperative pain management. Enhancing patient satisfaction and facilitating smooth rehabilitation after surgery to enable a quick return to daily life are crucial. Since the effectiveness of local anesthetic infiltration was first reported by Mullen et al. in lumbar spine surgery, this approach has been widely utilized. However, research on the use and efficacy of this technique in cervical spine surgery is lacking. Therefore, this study on the effects and safety of multimodal cocktail injection after cervical laminoplasty is significant. The study is expected to provide insights into the potential utility of multimodal cocktail analgesic injection in spinal surgery overall.

**8. Criteria for Discontinuation/Dropout**

Study subjects can discontinue the trial at any time by their request or at the discretion of the investigator due to safety, administrative reasons, or other reasons. The investigator should inquire about the reason for discontinuation, request the subject's final visit, and make efforts to observe the subject if there are unresolved adverse reactions.

The trial may be discontinued early in the following cases:

1. If the study subject withdraws consent for participation.
2. Violation of selection/exclusion criteria.
3. Major violation of the clinical trial plan.
4. Issues with administering the investigational drug to the study subject.
5. Difficulty in continuing the trial due to adverse reactions.
6. Need for the administration of concomitant drugs affecting safety/efficacy evaluation or deemed necessary for the study subject.
7. Inability to observe the study subject for follow-up.
8. Investigator deems the continuation of the trial inappropriate for the study subject.
9. Discovery of pregnancy by a female study subject during the administration of the investigational drug.

**9. Criteria for Effect Evaluation, Evaluation Methods, and Interpretation Methods (Statistical Analysis Methods)**

1. Statistically compare VAS scores for pain after surgery between the two groups.
2. Additionally, statistically compare Visual Analog Scale (VAS) scores, opioid consumption, rescue analgesic consumption, adverse effects, NDI, and JOA scores.

**10. Risks Expected for Subjects in This Study**

Possible Side Effects/Adverse Reactions:

(1) Ropivacaine

1) Cardiovascular: Hypertension, bradycardia, chest pain, hypotension, syncope

2) Central Nervous System: Fever, headache, dizziness, chills, anxiety, drowsiness

3) Dermatological: Pruritus

4) Metabolic/Endocrine: Hypokalemia

5) Genitourinary: Urinary retention, urinary tract infection

6) Hematological: Anemia

7) Neuromuscular: Abnormal sensation, sensory impairment, stiffness, perioral sensation abnormalities

8) Renal: Decreased urine output

9) Respiratory: Difficulty breathing

10) Other: Tremors

(2) Triamcinolone

1) Skin: Itching, allergic contact dermatitis, dryness, folliculitis, erythema, skin atrophy, striae, urticaria, mucosal atrophy

2) Local: Burning sensation, irritation

(3) Epinephrine

1) Cardiovascular: Angina, palpitations, chest pain, flushing, hypertension, myocardial oxygen consumption increase, pallor, cardiac arrhythmia, sudden death, bradycardia (injection), vasoconstriction, ventricular arrhythmia

2) Central Nervous System: Anxiety, dizziness, headache, insomnia, drowsiness, nervousness, syncope

3) Gastrointestinal: Dry mouth, nausea, vomiting, dry mouth

4) Genitourinary: Acute urinary retention in bladder outlet obstruction patients

5) Musculoskeletal: Weakness

6) Ophthalmic: Allergic conjunctival reactions, burning sensation, eye pain, eye irritation, angle-closure glaucoma exacerbation, transient myopia

7) Renal: Decreased renal or visceral blood flow

8) Respiratory: Difficulty breathing, wheezing

9) Other: Increased hair growth

(4) Ketorolac

1) Gastrointestinal: Dyspepsia, indigestion, abdominal pain, diarrhea, occasional constipation, abdominal bloating, vomiting, gastritis, rare gastrointestinal ulceration, gastrointestinal bleeding, perforation, transient elevations of liver enzymes

2) Systemic: Occasionally edema, rare anaphylaxis, bronchospasm, laryngeal edema, tongue edema, hypotension, flushing, hypersensitivity reactions such as rash, weight gain, fever, infection, malaise

3) Cardiovascular: Hypertension, rare flushing, cardiac arrhythmia, pallor, hypotension, fainting, syncope, chest pain, heart failure may occur

4) Dermatological: Rash, itching occasionally, toxic epidermal necrolysis (Lyell's syndrome), Stevens-Johnson syndrome, exfoliative dermatitis, urticaria, angioedema, flushing may occur

5) Hematologic and Lymphatic: Agranulocytosis, occasionally surgical site bleeding, thrombocytopenia, melena, neutropenia, eosinophilia, hematoma may occur

6) Nervous System: Headache, warmth occasionally, drowsiness, dizziness occasionally, rare convulsions, progression, hallucinations, abnormal dreams, abnormal sensation, perception disorder, depression, insomnia, nervousness, pruritus, severe thirst, abnormal thoughts, concentration disorder, excessive motor activity, confusion, muscle pain, aseptic meningitis (especially in patients with autoimmune diseases such as systemic lupus erythematosus (SLE) and mixed connective tissue disease (MCTD)), anxiety, psychotic reactions may occur

7) Hepatic: Rarely hepatitis, hepatic insufficiency, cholestatic jaundice, abnormal liver function tests may occur, and if these symptoms occur, administration should be discontinued immediately

8) Respiratory: Occasionally dyspnea, asthma, pulmonary edema, rhinitis, cough may occur

9) Urinary System: Rare acute renal failure, occasional lower abdominal pain (may be accompanied by hematuria and hyperazotemia), polyuria, nephritis, dysuria, oliguria, urinary retention, hemolytic uremic syndrome, hyperkalemia, hyponatremia, nephrotic syndrome, increased serum uric acid and creatinine levels may occur

10) Other: Occasionally injection site pain, taste disturbance, visual disturbance, blurred vision, optic neuritis, tinnitus, hearing loss, infertility in females, prolonged bleeding time may occur.

(5) Cefotiam

1) Shock: Rarely may cause shock, so observe carefully and discontinue administration and take appropriate measures if discomfort, oral abnormalities, tinnitus, dizziness, vomiting, etc., occur.

2) Hypersensitivity Reaction: Occasionally rash, urticaria, erythema, pruritus, fever, lymph node swelling, joint pain, etc., may occur; discontinue administration and take appropriate measures if these symptoms occur.

3) Skin: Rarely Stevens-Johnson syndrome, toxic epidermal necrolysis may occur; observe carefully and discontinue administration if abnormalities are recognized.

4) Hematological: Occasionally anemia, leukopenia, decreased red blood cells, increased eosinophils, decreased platelets, rarely pancytopenia may occur; discontinue administration and take appropriate measures if abnormalities are recognized.

5) Hepatic: Rarely elevated AST, ALT, ALP, jaundice, elevated LDH, γ-GTP may occur; observe carefully and discontinue administration if abnormalities are recognized.

6) Renal: Rarely severe renal impairment such as acute renal failure may occur; observe carefully and take appropriate measures if abnormalities are recognized.

7) Gastrointestinal: Rarely severe colitis with bloody stools may occur. Discontinue administration immediately if symptoms such as abdominal pain and frequent diarrhea occur.

8) Respiratory: Rarely pneumonia with seizures, eosinophilic lung infiltration may occur with symptoms such as fever, cough, difficulty breathing, and increased eosinophils. Discontinue administration and provide appropriate treatment such as corticosteroid administration if such symptoms occur.

9) Central Nervous System: Seizures may occur in patients with renal failure when administered in large doses.

10) Fungal Overgrowth: Rarely can cause colitis, candidiasis.

11) Vitamin Deficiency: Rarely vitamin K deficiency symptoms (hypoprothrombinemia, bleeding tendency, etc.), vitamin B group deficiency symptoms (glossitis, stomatitis, anorexia, neuropathy, etc.) may occur.

12) Other: Dizziness, headache, malaise, numbness may occur rarely.

(6) Morphine

1) Dependence: Continuous administration may lead to drug dependence, so observe carefully and administer cautiously. If symptoms such as yawning, sneezing, tearing, sweating, goosebumps, vomiting, diarrhea, abdominal pain, pupillary dilation, headache, insomnia, anxiety, hallucinations, seizures, tremors, muscle and joint pain, respiratory distress, and arrhythmia appear when reducing the daily dose gradually, observe the patient's condition carefully.

2) Respiratory depression: Symptoms such as difficulty breathing, slow breathing, irregular breathing, and apnea may occur due to respiratory depression, so observe carefully. If symptoms occur, administer a narcotic antagonist (naloxone, etc.) and provide appropriate treatment such as respiratory support. When administered intravenously, the maximum central nervous system effect is delayed by about 30 minutes, so rapid administration may lead to overdose. When rapidly injected into the epidural or subarachnoid space, the drug can redistribute directly to the brain respiratory center, causing early respiratory depression, which may be delayed up to 24 hours. Respiratory depression was more frequently observed with subarachnoid administration than with epidural administration, but most cases involved much higher doses than the recommended dose.

3) Brain Injury and Increased Intracranial Pressure: Secondary increase in cerebrospinal fluid pressure due to the respiratory depression action of this drug may worsen head injury, other intracranial lesions, or pre-existing increased intracranial pressure. Additionally, narcotic analgesics can obscure the clinical course of patients with head injuries.

4) Hypotension and Shock: Narcotic analgesics, including this drug, may cause severe hypotension and shock in patients with reduced blood pressure due to hypovolemia or concomitant use of phenothiazine drugs, general anesthetics, etc. Vascular dilation induced by this drug may further reduce cardiac output and blood pressure, so administer with caution in patients with circulatory shock.

5) Muscle Rigidity in the Legs: Muscle rigidity was reported in patients who received subarachnoid administration exceeding 20 mg per day.

6) Circulatory System: Low doses administered intravenously have little effect on cardiovascular stability, but high doses can cause convulsions due to increased sympathetic nervous system activity and excitement caused by increased circulation catecholamines.

7) Central Nervous System: Excitement of the central nervous system and seizures may occur with high-dose intravenous administration.

8) Psycho-Neurological: Discomfort, anxiety, addictive psychosis, hallucinations, confusion, headache, pain, hyperalgesia, and allodynia may occur.

9) Digestive System: Nausea and vomiting may occur, and symptoms can be relieved by administering a low dose (0.2 mg) of naloxone. Constipation, biliary colic, and dry mouth may occur.

10) Urinary System: In about 90% of male patients, urinary retention persisted for 10 to 20 hours after a single epidural or subarachnoid administration, but the incidence was lower in females. Occasionally, catheter insertion may be necessary, and caution should be exercised for adverse reactions (e.g., sepsis) during catheter insertion. Symptoms can be relieved by administering a low dose (0.2 mg) of naloxone. Urinary retention may occur.

11) Skin: Itching may occur, and it is related to the dose administered and is independent of the administration site. Symptoms can be relieved by administering a low dose (0.2 mg) of naloxone.

12) Hypersensitivity Reactions: Hives and local tissue irritation reactions may occur.

13) Other: Cough reflex suppression, decreased libido in both men and women, irregular menstruation, amenorrhea, and thermoregulation disorders may occur.

14) Gastrointestinal Tract: Intestinal obstruction.

**11. Evaluation of Safety, Including Side Effects, and Reporting Methods**

Record and evaluate side effects between groups and report them.

**12. Information and Management of Investigational Medicinal Products for Clinical Trials**

Investigational Medicinal Products

(1) Ropiva Injection (ROPIVA Inj. Hanlim Pharm Co., Ltd)

1) Composition and Dosage: Ropivacaine hydrochloride hydrate 7.9 mg/mL

2) Form and Appearance: Injection

3) Storage: Sealed container, store at room temperature (1-30℃)

4) Manufacturer: Hanlim Pharmaceutical

5) Usage/Dosage: 3 times a day, orally administered 1 tablet per dose

(2) Tamceton Injection (TAMCETON 40 INJ. Hanall Biopharma)

1) Composition and Dosage: Triamcinolone Acetonide 40mg/mL

2) Form and Appearance: Injection

3) Storage: Light-resistant sealed container, store at room temperature (1-30℃)

4) Manufacturer: Hanall Biopharm

(3) Epinephrine Injection (EPINEPHRINE DAIHAN INJ. Daihan Pharm Co., Ltd)

1) Composition and Dosage: Epinephrine 1mg/mL

2) Form and Appearance: Injection

3) Storage: Light-resistant sealed container, store below 25℃, do not refrigerate

4) Manufacturer: Daehan Pharmaceutical

(4) Ketocin Injection (KETOCIN INJ 30mg/ml. Myungmoon Pharm. Co., Ltd)

1) Composition and Dosage: Ketorolac tromethamine 30 mg/mL

2) Form and Appearance: Injection

3) Storage: Light-resistant sealed container, store at room temperature (1-30℃)

4) Manufacturer: Myungmoon Pharmaceutical

(5) Jetiam Injection (JETIAM INJ 1g. Samjin Pharm)

1) Composition and Dosage: Cefotiam Hydrochloride-Dried Sodium Carbonate 1.242g

2) Form and Appearance: Injection

3) Storage: Sealed container, store at room temperature (1-30℃)

4) Manufacturer: Samjin Pharmaceutical

(6) BC Morphine Sulfate Injection (BC MORPHINE SULFATE INJ 1mg/mL)

1) Composition and Dosage: Morphine Sulfate hydrate 1mg/mL

2) Form and Appearance: Injection

3) Storage: Light-resistant sealed container, store at room temperature (1-30℃)

4) Manufacturer: BC World Pharmaceutical

**13. Informed Consent Form**

An informed consent form is required for research involving postoperative pain evaluation.

**14. Regulations for Victim Compensation**

See attached document.

**15. Case Record Form Template**

See attached document.

**16. Criteria for Treatment and Care of Subjects After Clinical Trials**

Typically, a questionnaire assessing changes in symptoms during outpatient visits is conducted for patients with cervical spinal stenosis and peripheral neuropathy.

**17. Measures for Subject Safety Protection**

Subject information is not disclosed anywhere, and subjects are coded by gender, age, and number sequence, recording only the results. The investigator must conduct the clinical trial in accordance with the Declaration of Helsinki, considering the rights and welfare of the subjects. Participants in this clinical trial should be familiar with the drug clinical trial management standards and the clinical trial protocol. The investigator thoroughly evaluates the suitability of each subject and the occurrence of adverse reactions through interviews and examinations, allocating sufficient time for each subject. Collaborating researchers regularly report on adverse reactions, trial progress, situation, and results to the principal investigator, who manages the clinical trial progress periodically.

**18. Data Recording and Storage Method**

Documents related to clinical research are transferred to the document storage responsibility person at the institute after the end of the study and are stored for three years.

**19. Audit and Monitoring Plan**

To ensure the rights and welfare protection of subjects and verify whether clinical trial-related data matches the documented evidence and is accurate, complete, and verifiable, the principal investigator may request an audit or monitoring from the Institutional Review Board for Clinical Trials, or actively cooperate with audits conducted by the board.

**References**

1. Sakai Y, Matsuyama Y, Inoue K, Ishiguro N. Postoperative instability after laminoplasty for cervical myelopathy with spondylolisthesis. J Spinal Disord Tech. 2005;18(1):1-5.

2. Kimura A, Seichi A, Inoue H, Hoshino Y. Long-term results of double-door laminoplasty using hydroxyapatite spacers in patients with compressive cervical myelopathy. Eur Spine J. 2011;20(9):1560-6.

3. Cho SK, Kim JS, Overley SC, Merrill RK. Cervical Laminoplasty: Indications, Surgical Considerations, and Clinical Outcomes. J Am Acad Orthop Surg. 2018;26(7):e142-e52.

4. Weinberg DS, Rhee JM. Cervical laminoplasty: indication, technique, complications. J Spine Surg. 2020;6(1):290-301.

5. Trousdale RT, McGrory BJ, Berry DJ, Becker MW, Harmsen WS. Patients' concerns prior to undergoing total hip and total knee arthroplasty. Mayo Clin Proc. 1999;74(10):978-82.

6. Park KK, Shin KS, Chang CB, Kim SJ, Kim TK. Functional disabilities and issues of concern in female Asian patients before TKA. Clin Orthop Relat Res. 2007;461:143-52.

7. Capdevila X, Barthelet Y, Biboulet P, Ryckwaert Y, Rubenovitch J, d'Athis F. Effects of perioperative analgesic technique on the surgical outcome and duration of rehabilitation after major knee surgery. Anesthesiology. 1999;91(1):8-15.

8. Morrison SR, Magaziner J, McLaughlin MA, Orosz G, Silberzweig SB, Koval KJ, et al. The impact of post-operative pain on outcomes following hip fracture. Pain. 2003;103(3):303-11.

9. Deng Z, Li Y, Storm GR, Kotian RN, Sun X, Lei G, et al. The efficiency and safety of steroid addition to multimodal cocktail periarticular injection in knee joint arthroplasty: a meta-analysis of randomized controlled trials. Sci Rep. 2019;9(1):7031.

10. Chelly JE, Ben-David B, Williams BA, Kentor ML. Anesthesia and postoperative analgesia: outcomes following orthopedic surgery. Orthopedics. 2003;26(8 Suppl):s865-71.

11. Kehlet H, Dahl JB. The value of "multimodal" or "balanced analgesia" in postoperative pain treatment. Anesth Analg. 1993;77(5):1048-56.

12. Burroughs TE, Davies AR, Cira JC, Dunagan WC. Understanding patient willingness to recommend and return: a strategy for prioritizing improvement opportunities. Jt Comm J Qual Improv. 1999;25(6):271-87.

13. Brokelman RB, van Loon CJ, Rijnberg WJ. Patient versus surgeon satisfaction after total hip arthroplasty. J Bone Joint Surg Br. 2003;85(4):495-8.

14. Fisher CG, Belanger L, Gofton EG, Umedaly HS, Noonan VK, Abramson C, et al. Prospective randomized clinical trial comparing patient-controlled intravenous analgesia with patient-controlled epidural analgesia after lumbar spinal fusion. Spine (Phila Pa 1976). 2003;28(8):739-43.

15. Yukawa Y, Kato F, Ito K, Terashima T, Horie Y. A prospective randomized study of preemptive analgesia for postoperative pain in the patients undergoing posterior lumbar interbody fusion: continuous subcutaneous morphine, continuous epidural morphine, and diclofenac sodium. Spine (Phila Pa 1976). 2005;30(21):2357-61.

16. Jirarattanaphochai K, Jung S. Nonsteroidal antiinflammatory drugs for postoperative pain management after lumbar spine surgery: a meta-analysis of randomized controlled trials. J Neurosurg Spine. 2008;9(1):22-31.

17. Wheeler M, Oderda GM, Ashburn MA, Lipman AG. Adverse events associated with postoperative opioid analgesia: a systematic review. J Pain. 2002;3(3):159-80.

18. Block BM, Liu SS, Rowlingson AJ, Cowan AR, Cowan JA, Jr., Wu CL. Efficacy of postoperative epidural analgesia: a meta-analysis. JAMA. 2003;290(18):2455-63.

19. Kurosaka K, Tsukada S, Seino D, Morooka T, Nakayama H, Yoshiya S. Local Infiltration Analgesia Versus Continuous Femoral Nerve Block in Pain Relief After Total Knee Arthroplasty: A Randomized Controlled Trial. J Arthroplasty. 2016;31(4):913-7.

20. Li D, Tan Z, Kang P, Shen B, Pei F. Effects of multi-site infiltration analgesia on pain management and early rehabilitation compared with femoral nerve or adductor canal block for patients undergoing total knee arthroplasty: a prospective randomized controlled trial. Int Orthop. 2017;41(1):75-83.

21. Nakai T, Nakamura T, Nakai T, Onishi A, Hashimoto K. A study of the usefulness of a periarticular multimodal drug cocktail injection for pain management after total hip arthroplasty. J Orthop. 2013;10(1):5-7.

22. Salwan A, Pisulkar GL, Taywade S, Awasthi AA, Saoji A, Jadawala VH, et al. A Review on the Efficacy of Extraosseous Local Infiltration of Multimodal Drug Cocktail for Pain Management After Total Knee or Hip Arthroplasty. Cureus. 2022;14(10):e30451.

23. Perera AP, Chari A, Kostusiak M, Khan AA, Luoma AM, Casey ATH. Intramuscular Local Anesthetic Infiltration at Closure for Postoperative Analgesia in Lumbar Spine Surgery: A Systematic Review and Meta-Analysis. Spine (Phila Pa 1976). 2017;42(14):1088-95.

24. Kraiwattanapong C, Arnuntasupakul V, Kantawan R, Woratanarat P, Keorochana G, Langsanam N. Effect of Multimodal Drugs Infiltration on Postoperative Pain in Split Laminectomy of Lumbar Spine: A Randomized Controlled Trial. Spine (Phila Pa 1976). 2020;45(24):1687-95.

25. Elder JB, Hoh DJ, Liu CY, Wang MY. Postoperative continuous paravertebral anesthetic infusion for pain control in posterior cervical spine surgery: a case-control study. Neurosurgery. 2010;66(3 Suppl Operative):99-106; discussion -7.

26. Southerland WA, Gillis J, Urits I, Kaye AD, Eskander J. Intraoperative Administration of Dexmedetomidine and Dexamethasone in Local Anesthetic Infiltration to Improve Postoperative Pain Control After Posterior Cervical Fusion. Cureus. 2021;13(4):e14699.

27. Bianconi M, Ferraro L, Ricci R, Zanoli G, Antonelli T, Giulia B, et al. The pharmacokinetics and efficacy of ropivacaine continuous wound instillation after spine fusion surgery. Anesth Analg. 2004;98(1):166-72.

28. Bajwa SJ, Haldar R. Pain management following spinal surgeries: An appraisal of the available options. J Craniovertebr Junction Spine. 2015;6(3):105-10.

29. Mullen JB, Cook WA, Jr. Reduction of postoperative lumbar hemilaminectomy pain with Marcaine. Technical note. J Neurosurg. 1979;51(1):126-7.

30. Kjaergaard M, Moiniche S, Olsen KS. Wound infiltration with local anesthetics for post-operative pain relief in lumbar spine surgery: a systematic review. Acta Anaesthesiol Scand. 2012;56(3):282-90.
